# Trichilianones A-D, Novel Cyclopropane-Type Limonoids from *Trichilia adolfi*

**DOI:** 10.3390/molecules26041019

**Published:** 2021-02-15

**Authors:** Ivan Limachi, Mariela Gonzalez-Ramirez, Sophie Manner, Juan C. Ticona, Efrain Salamanca, Alberto Gimenez, Olov Sterner

**Affiliations:** 1 Department of Chemistry, Centre for Analysis and Synthesis, Lund University, 22100 Lund, Sweden; ivan.limachi_valdez@chem.lu.se (I.L.); mariela_alejandra.gonzalez_ramirez@chem.lu.se (M.G.-R.); sophie.manner@chem.lu.se (S.M.); 2 Instituto de Investigaciones Farmaco Bioquimicas, Universidad Mayor de San Andres, La Paz, Bolivia; biojuancarlos_11@yahoo.com (J.C.T.); efrain_salamanca@hotmail.com (E.S.); agimenez@megalink.com (A.G.)

**Keywords:** *Trichilia adolfi*, cyclopropane-limonoids, cytotoxicity, leishmanicidal activity

## Abstract

The fractionation of an ethanol extract of the bark of *Trichilia adolfi* yielded four novel limonoids (trichilinones A-D, **1**–**4**), with five fused rings and related to the hortiolide-type limonoids. Starting with an ε-lactone, which is α,β-unsaturated in trichilinones A and D (**1** and **4**), attached to a tetrahydrofuran ring that is connected to an unusual bicyclo [5.1.0] hexane system, joined with a cyclopentanone with a 3-furanyl substituent [(2-oxo)-furan-(5*H*)-3-yl in trichilinone D (**4**)], the four compounds isolated display a new 7/5/3/5/5 limonoid ring system. Their structures were established based on extensive analysis of NMR spectroscopic data. As the crude extract possessed anti-leishmanial properties, the compounds were assayed for cytotoxic and anti-parasitic activities in vitro in murine macrophages cells (Raw 264.7) and leishmania promastigotes (*L. amazoniensis* and *L. braziliensis*), respectively. The compounds showed moderate cytotoxicity (approximately 70 μg/mL), but are not responsible for the leishmanicidal effect of the extract.

## 1. Introduction

The genus *Trichilia* is vast, comprising flowering plants belonging to the Meliaceae family. Additionally included in *Trichilia* are trees widely distributed across the tropical forests in America, Europe, Africa and Asia [1]. In South America, *Trichilia* species are used in traditional medicine by several cultures throughout the continent, for example, in Bolivia, where this has been conserved in the traditional medicine of the Tacana [2]. An ethnobotanical report reviewing the medicine of the Tacana identified three *Trichilia* species, *T. inaeqilatera*, *T. pleeanea* and *T. adolfi*, as plants that have been used to treat lung, kidney and liver pains [3]. The treatment consists of preparing an infusion of the bark boiled in water, which was drunk daily and the procedure was widespread among the Tacanas. During an ongoing screening of Bolivian plants for metabolites with antiparasitic activities [4], an ethanol extract of the bark of *T. adolfi* was found to possess antileishmanial properties. As this species is poorly investigated for contents of secondary metabolites, a phytochemical study including the testing of isolated metabolites for antileishmanial activities, motivated the examination presented here.

**Figure 1:**
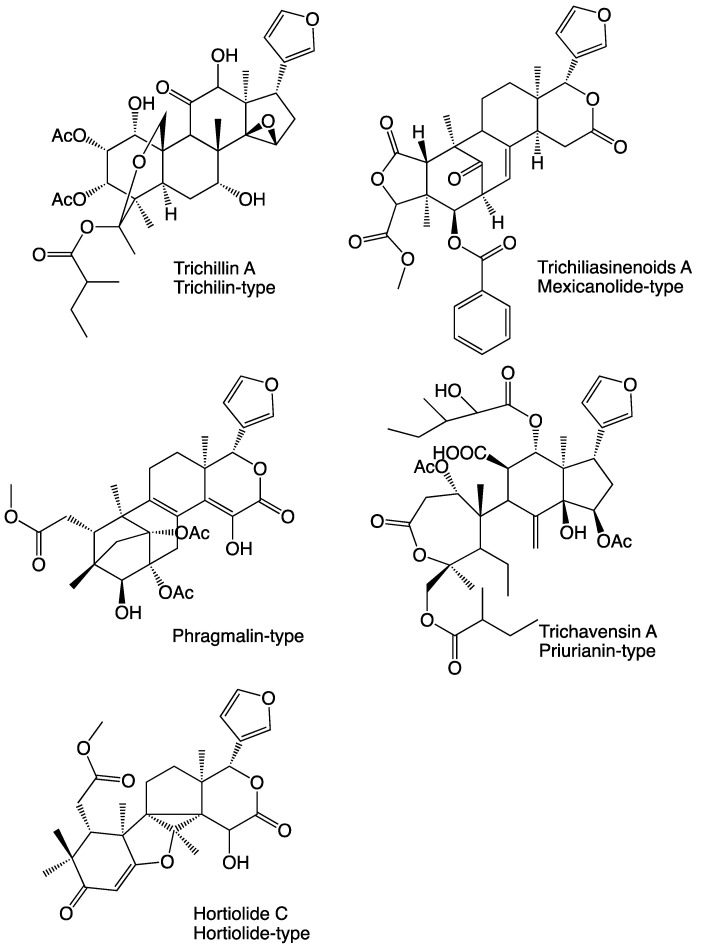
Related compounds isolated from other sources.

The secondary metabolites of other *Trichilia* species have been studied, and a large number of limonoids and other terpenoids have been isolated. To some extent, these were investigated in various biological assays. Limonoids are compounds based on the triterpenoid class, and as a family of phytochemicals, they display a huge diversity. Structurally, they are highly oxygenated tetranortriterpenoids with various carbon skeleta, normally possessing furan and lactone rings, and structurally classified by their basic four rings [5] as well as modifications in these. The most representative examples found in the genus *Trichilia* are the trichilin-type limonoids [6] that conserve the four-ring system of the triterpene precursor intact, the mexicanolide-type limonoids [7], the phragmalin-type limonoids [8], and the prieurianin-type limonoids [9]. Examples of these are shown in Figure 1.

An initial focus was to use limonoids as bio-pesticides, and a well-known example that received much attention is the potent antifeedant azadirachtin, isolated from the seeds of the neem tree (*Azadirachta indica*) [10]. However, recent studies are more oriented towards their antibiotic [11], cytotoxic [12] and anticancer [13] properties.

The present study is focused on the characterization of metabolites present in an extract of the bark of *Trichilia adolfi* (see Experimental section for details). The structures of the isolated metabolites were elucidated after an extensive spectroscopic analysis based essentially on the elemental composition determined by high-resolution mass spectrometry and 1D NMR as well as 2D NMR experiments. The cytotoxicity of pure compounds was evaluated in vitro in cell cultures of murine macrophages (Raw 264.7), while the leishmanicidal properties were evaluated in the two leishmania-promastigote strains *Leishmania amazoniensis* and *L. braziliensis* (see Experimental section for details).

## 2. Results and Discussion

**Figure 2:**
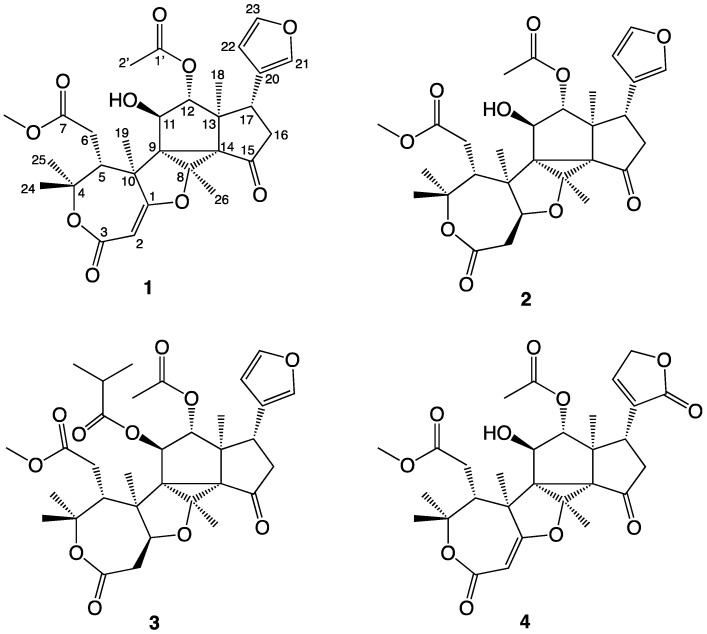
Trichilianones A (**1**), B (**2**), C (**3**) and D (**4**), isolated from Trichilia adolfi.

**Figure 3:**
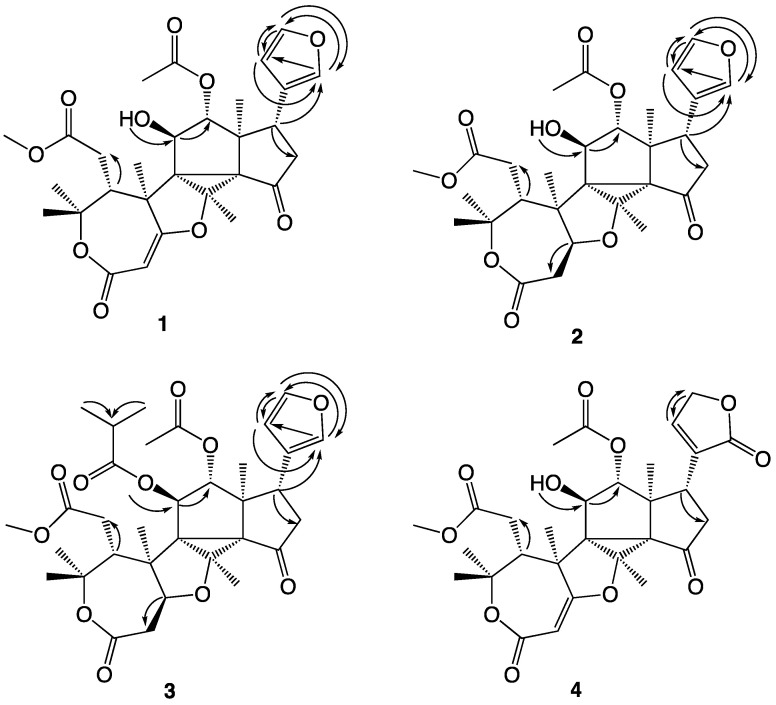
COSY correlations observed with compounds **1**–**4**.

**Figure 4:**
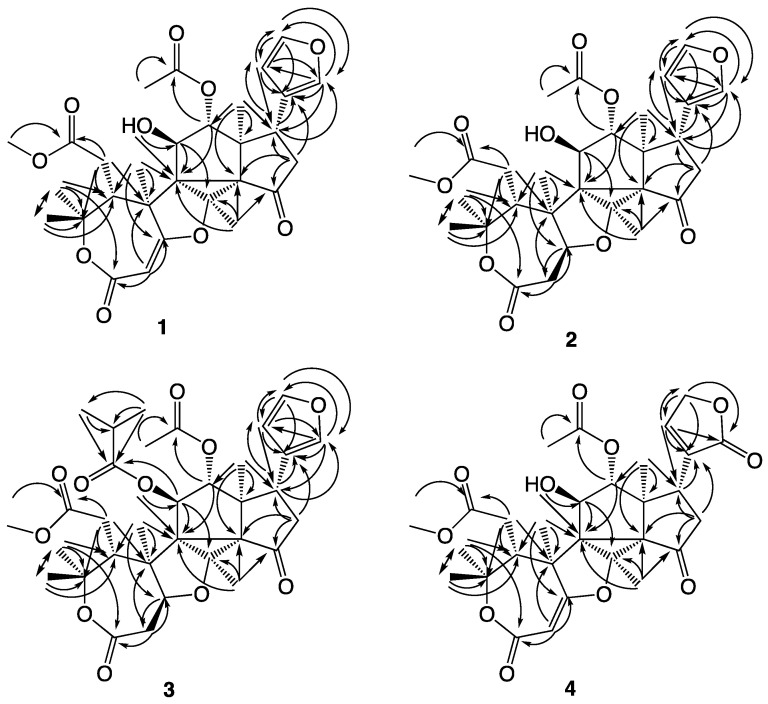
HMBC correlations observed with compounds **1**–**4**.

**Figure 5:**
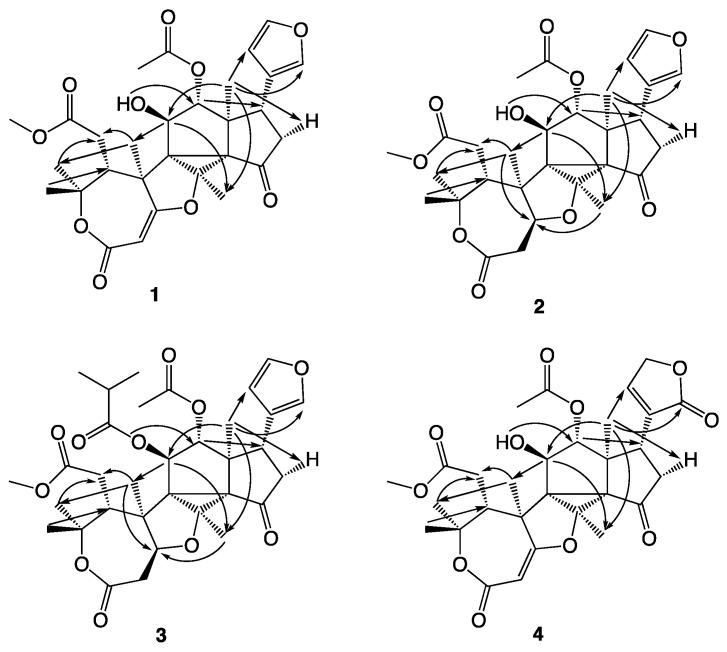
NOESY correlations observed with compounds **1**–**4**.

The structures of the novel compounds isolated are shown in Figure 2, while the ^1^H and ^13^C NMR data are given in Table 1 and Table 2. The COSY correlations observed for compounds **1**–**4** are summarized in Figure 3, the HMBC correlations in Figure 4, and the NOESY correlations in Figure 5.

### 2.1. Structure Determination of Trichilianone A *(****1****)*

Trichilianone A (**1**, name proposed by us and derived from the plant genus name Trichilia) was isolated as a white amorphous powder, although repeated re-crystallization in methanol/water (1:2) eventually gave crystals with the melting point 181 °C. Sadly, any attempt to obtain an X-ray structure of these failed, however, the elemental composition of **1** could be determined by LC-HRMS experiments. The [M + H]^+^ ion was observed at *m*/*z* 543.2252 which corresponds to C_29_H_35_O_10_ (calculated 543.2230). This is in accordance with the 1D ^1^H and ^13^C NMR data (^13^C spectra as both protons decoupled as well as DEPT spectra). See Table 1 and Table 2 for NMR data, SI for spectra, and Figure 3, Figure 4 and Figure 5 for 2D NMR correlations. The 1D ^1^H and ^13^C NMR display signals for 34 protons, of which one is exchangeable, and 29 carbons. The unsaturation index of **1** is consequently 13, and as the NMR data suggest, the presence of four carbonyl groups and three carbon–carbon double bonds hints that **1** contains six rings. In addition, the 1D NMR data indicate the presence of twelve non-protonated carbons, of which one is a ketone and three are ester or lactone carbonyls, eight methine groups of which four appear to be olefinic while two are saturated but oxygenated, two methylene groups, and seven methyl groups of which one is a methoxy group. The elucidation of the structure of **1** required extensive 2D NMR analyses, during which the COSY, HMQC, HMBC and NOESY NMR spectra proved to be essential. The atom numbering system (see Figure 2) used in this study follows that proposed earlier in the report on the hortiolide limonoids isolated from *Hortia colombiana* [14].

As is often the case when determining the structures of terpenoids, the observation of HMBC correlations from the methyl protons to the adjacent carbons gives a lot of information. The correlation of the methoxy protons at 3.67 ppm to C-7 at 174.4 ppm suggests that this methyl group is part of a methyl ester. 2′-H_3_ (2.13 ppm), as well as 12-H (4.91 ppm), correlate to C-1′ (172.0 ppm), suggesting that C-1′ and C-2′ are part of an acetyloxy group connected to C-12 (which with the carbon shift 83.1 ppm is expected to be oxygenated). 18-H_3_ (0.95 ppm) give correlations to C-12, C-17, C-13 and C-14, of which the two latter are non-protonated, while 19-H_3_ (1.67 ppm) give correlations to C-1, C-5, C-9 and C-10. The two methyl groups 24-H_3_ and 25-H_3_ both give strong HMBC correlations to each other carbon, showing that they are geminal, as well as to C-4 and C-5. They are obviously positioned at the oxygenated C-4 (83.1 ppm), and the correlation to C-5 connected 24-H_3_ and 25-H_3_ with 19-H_3_. Finally, 26-H_3_ give strong HMBC correlations to C-8, C-9 and C-14, all non-protonated carbons, showing that C-9 is the link between 19-H_3_ and 26-H_3_ and that C-14 is the link between 18-H_3_ and 26-H_3_. These connectivities demonstrate that the 18-H_3_ methyl group is attached to C-13, and that the 19-H_3_ methyl group is attached to C-10. In addition, the fact that 26-H_3_ only gives HMBC correlations to three carbons indicates that it is positioned on an oxygenated carbon, which consequently must be C-8 (at 77.5 ppm).

With this partial skeleton at hand, we proceeded with the positioning of additional groups and the formation of the six rings. The COSY coupling between 5-H and 6-H_2_ together with the HMBC couplings from 6-H_2_ to C-4, C-5, C-7 and C-10 establishes the link C-5/C-6/C-7, with C-7 as indicated above as the carbonyl carbon in a methyl ester. Careful inspection of the HMBC data revealed a relatively weak but clear correlation between 25-H_3_ and C-3, and as 2-H gives HMBC correlations to C-1, C-3 and C-10 the C-1/C-2 carbon-carbon double bond is part of the first ring (an ε-lactone). No HMBC correlation between 2-H and a third carbon substituent of this double bond was observed, suggesting that C-2 is connected to oxygen. This is supported by the extreme ^13^C chemical shifts of the two double bond carbons, 94.2 and 180.2 ppm, as well as the short-range ^1^H-^13^C coupling [^1^*J*(C,H)] constant of 173 Hz between 2-H and C-2 (C,H-2) (observed in the HMBC spectrum). The ε-lactone is therefore not only α,β-conjugated but also substituted with oxygen in the β-position. Similar ε-lactones are found in the toonayunnanin-type limonoids reported from the genus *Toona* (Meliaceae) [15].

Continuing to the other end of the molecule, we find a substituent that readily can be identified as a 3-furanyl ring, by the NMR chemical shifts, coupling constants, as well as the COSY and HMBC correlations. 21-H (7.33 ppm, ddd, *J* = 0.7, 0.9, 1.6 Hz) couples in the COSY spectrum with 17-H, 22-H and 23-H, and in the HMBC spectrum with C-17, C-20, C-22 and C-23. 22-H (6.30 ppm, dd, *J* = 0.9, 1.8 Hz) correlates with 21-H and 23-H (COSY) and with C-17, C-20, C-21 and C-23 (HMBC), while 23-H (7.44 ppm, dd, *J* = 1.6, 1.8 Hz) correlates with 21-H and 22-H (COSY) and with C-20, C-21 and C-22 (HMBC). Additionally, the ^1^*J*(C,H) coupling constants of C,H-21 (200 Hz), C,H-22 (173 Hz) and C,H-23 (203 Hz) observed in the HMBC spectrum are consistent with a 3-furanyl moiety. The furanyl group appears to be linked to C-17, and this is confirmed by the HMBC correlations from 17-H to C-20, C-21 and C-22. 3-Furanyl substituents are common in limonoids, and comparing the NMR data with the terpenoids reported from *Trichilia havanensis* [16] confirm that the second ring of **1** is a furan. The strong ^1^H-^1^H coupling between 17-H and 16-H_2_ connects C-17 with C-16, and HMBC correlations from 16-H_2_ to C-15 connect C-16 to C-15. HMBC correlations can also be observed from 16-H_2_ to C-14, C-17 and C-20. Together with a relatively weak but clear HMBC correlation from 26-H_3_ to C-15, C-14 is connected to C-15, which establishes the third ring as a cyclopentanone. 

Thereby, only one carbon (C-11) remains to be inserted into the structure, although in a way that forms the three remaining rings. C-11 is an oxygenated methine, but unlike C-12, its oxygen is not functionalized but remains a free hydroxyl group. The 11-OH proton signal can be observed in the ^1^H NMR spectrum at 3.66 ppm as a doublet (2.6 Hz), it couples to 11-H in the COSY spectrum and gives HMBC correlations to C-9 and C-11. The ^1^H-^1^H coupling between 11-H and 12-H suggests that C-11 is situated between C-9 and C-12, and this is confirmed by the HMBC correlations between 11-H, 11-OH as well as 12-H and C-9, as well as between 11-H and C-8. In the discussion above, a conclusion was that C-1 and C-8 are oxygenated, and as nine of the molecule’s ten oxygens were already used, only one alternative remains. The tenth oxygen has to be placed between C-1 and C-8, forming a tetrahydrofuran ring. Thereby all 29 carbons, 34 hydrogens and 10 oxygens are accounted for, and the only way to complete the structure is to form a bond between the two non-protonated carbons C-9 and C-14 and thereby also forming the two last rings of the molecule. Furthermore, **1** has an unusual carbon skeleton with an uncommon combination of rings. Similarities can be seen with the hortiolide-type limonoids [17] (see Figure 1) isolated from species belonging to the genus *Hortia*, of which some possess the cyclopentan-cyclopropane-tetrahydrofuran ring combination present in the central part of **1**. 

The relative configuration of **1** was determined from correlations observed in NOESY experiments. 18-H_3_ correlate to 11-H, 16-Hb, 21-H, 22-H and 26-H_3_, and 11-H also shows correlations with 26-H_3_ as well as 19-H_3_. A correlation is observed between 12-H and 17-H, suggesting that these two protons are on the opposite side of the molecule compared to 18-H_3_, 19-H_3_, 11-H, 16-Hb, 26-H_3_ as well as the furan ring. This is confirmed by the NOESY correlation between 11-OH and 12-H. 19-H_3_ show correlations with 6-H_2_ and 25-H_3_, also 25-H_3_ correlate to 6-H_2_ while 24-H_3_ correlate with 5-H, demonstrating the relative configuration around the ε-lactone ring. The corresponding configuration was suggested in the hortiolide-type limonoids [17].

### 2.2. Structure Determination of Trichilianone B (***2***)

Trichilianone B (**2**) was isolated as a colourless amorphous powder, for which HRMS experiments suggested the elemental composition C_29_H_36_O_10_, (*m*/*z*: 545.2395 [M + H]^+^, calculated for C_29_H_37_O_10_ 545.2387). This is in accord with the 1D NMR spectra (see Table 1 and Table 2 for data, and SI for NMR spectra), which show the presence of 29 carbons and 35 protons, corresponding to 12 degrees of unsaturation that is one less compared to **1**. The 35 protons all show correlations to carbons in the HMQC spectrum, indicating that the 36th is exchangeable and not visible in the proton spectrum. From the 1D NMR spectra, it is evident that a major difference between compounds **1** and **2** appears to have taken place around the conjugated enol ether (C-1 and C-2). The carbon signal of C-2 appears at 94.2 ppm in **1**, and although there are two similar resonances of protonated carbons in the ^13^C NMR spectrum of **2** (at 90.8 and 91.2 ppm), their ^1^*J*(C,H) coupling constants (148 and 151 Hz) do not indicate that they are part of a conjugated enol ether. This suggests that the C-1/C-2 double bond of **1** is reduced in **2**, accounting for the loss of a degree of unsaturation, and this was confirmed by the following NMR analyses.

As with **1**, the major part of the carbon skeleton is revealed by the HMBC correlations from the methyl protons to the adjacent carbons. The presence of a methyl ester was shown by the correlation from the methoxy protons to C-7, and a C-12 acetoxy group by the correlation of 2′-H_3_ (as well as 12-H) to C-1′. 18-H_3_ correlate with C-12, C-17, C-13 and C-14, the two former tertiary and the two latter quaternary, while 19-H_3_ give correlations to C-1, C-5, C-9 and C-10. The two geminal methyls 24-H_3_ and 25-H_3_ both give HMBC correlations to C-4 and C-5, as well as to themselves, and are consequently both positioned on the oxygenated C-4. As 26-H_3_ gives strong HMBC correlations to the non-protonated carbons C-8, C-9 and C-14, the same basic conclusions about the partial carbon skeleton as in **1** (*vide supra*) can be drawn for **2**. HMBC correlations from 2-H_2_ as well as from 25-H_3_ to C-3, together with the ^1^H-^1^H coupling between 1-H and 2-H_2_, close the ε-lactone ring, with, as predicted, a saturated C-1/C-2 bond in **2**. NMR signals and coupling constants corresponding to a 3-furanyl moiety attached to C-17 were observed in the spectra of **2** as well, the proton and carbon chemical shifts are quite similar, and the HMBC correlations from 17-H to C-13, C-16, C-20, C-21 and C-22 as well as from 16-H_2_ to C-15, C-17 and C-20 establish the presence of a cyclopentanone ring with a 3-furanyl substituent. The insertion of the final carbon, hydrogen and oxygen as a C-11 secondary alcohol function follows the reasoning for **1** (*vide supra*), although 11-OH was not observed in the proton spectrum. Finally, by forming the inevitable bond between C-9 and C-14 the remaining rings are formed, and the structure is complete. The determination of the relative configuration of **2** is discussed below.

### 2.3. Structure Determination of Trichilianone C (***3***)

Trichilianone C (**3**) is significantly bigger compared to **1** and **2**, and HRMS experiments suggested its elemental composition to be C_33_H_42_O_11_ (*m*/*z*: 615.2821 [M + H]^+^, calculated for C_33_H_43_O_11_ 615.2805). The signals for all 42 protons and 33 carbons could be observed in the ^1^H and ^13^C NMR spectra (see Table 1 and Table 2 for data, and SI for NMR spectra). Compared to **2**, the NMR data are in general similar, besides the additional signals for two methyl groups (C-3” and C-4”), a methine group (C-2”, forming an isopropyl group with the two methyls) and an ester or lactone carbonyl carbon (C-1”). A significant difference in the chemical shift of 11-H (4.33 ppm in **2** and 5.49 ppm in **3**) suggests that **3** simply is the C-11 isobutyric acid ester of **2**. As calculated from the elemental composition, the number of unsaturations in **3** has increased to 13, which is in accord with this suggestion. Nevertheless, a complete NMR structure elucidation of **3** was carried out, demonstrating that the structure is as proposed in Figure 1. The relative stereochemistry of **2** and **3** was determined by NOESY experiments, showing that they have a similar configuration as **1**. NOESY correlations were observed between 18-H_3_ and 11-H, 16-Hb, 21-H, 22-H as well as 26-H_3_, and 11-H also shows correlations with 26-H_3_ as well as 19-H_3_. A correlation is observed between 12-H and 17-H, suggesting that these two protons are on the opposite side of the molecule compared to 18-H_3_, 19-H_3_, 11-H, 16-Hb, 26-H_3_ as well as the furan ring. 19-H_3_ show correlations with 1-H, 6-H_2_ and 25-H_3_, also 25-H_3_ correlate to 6-H_2_ while 24-H_3_ correlates with 5-H, while 26-H_3_ correlates with 1-H. This demonstrates that the relative configurations of **2** and **3** are the same as determined for **1** and that the configuration of C-1 of **2** and **3** are as shown in Figure 2.

### 2.4. Structure Determination of Trichilianone D (***4***)

Trichilianone D (**4**, name proposed by us) is, through HRMS experiments, suggested to have the elemental composition C_29_H_34_O_11_ (*m*/*z*: 559.2171 [M + H]^+^, calculated for C_29_H_35_O_11_ 559.2179), almost like **1** but with one additional oxygen. Like **1**, **4** has 13 unsaturations, and the NMR data (see Table 1 and Table 2 for data and SI for spectra) indicate that the C-1/C-2 double bond is retained in **4** as a conjugated enol ether. As with **1**, the 11-OH proton can be observed in the NMR experiments, and it gives a COSY coupling to 11-H as well as HMBC couplings to C-9 and C-11. Major differences in the NMR data are observed for the signals of the 3-furanyl moiety of **1**, which appears to have been replaced by a ring containing oxygenated methylene (C-23), a trisubstituted carbon-carbon double bond (C-20/C-22), and a lactone carbonyl (C-21), in the form of a 2-oxo-furan-(5*H*)-3-yl moiety. Both 16-H_2_ and 17-H give HMBC correlations to C-20, 22-H strongly to C-21 and C-23 and weakly to C-17 and C-20, while 23-H_2_ correlate strongly to C-20 and C-22 and weakly to C-17 and C-21. The proton signal 23-H_2_ integrates for 2 protons, and both the proton and carbon chemical shifts are typical for an acyloxygenated methylene group (4.93 and 70.6 ppm, respectively). In addition, in the conjugated C-20/C-22 double bond the β-carbon show the expected elevated proton and carbon chemical shifts (7.32 and 148.7 ppm, respectively). The same oxidized 3-furanyl moiety has also been found in limonoids from other species [18].

Although this was not specifically studied in this investigation, it is reasonable to assume that the biosynthetic pathways leading to the trichilianones share critical steps with those producing the hortolides. The major difference between the two systems is that the cyclopentanone ring of the former was subjected to a Baeyer–Villiger-type oxidation in the latter, while the same type of oxidation introduced the ε-lactone functionality in the trichilianones. The mechanism for the formation of the cyclopropane ring, that both share, was studied, and a mechanism was suggested for hortionlide [17]. It has also been studied in diterpenoids from the thujane group [19]. In particular, in the trichilianones, this biosynthetic step introduces a considerable amount of ring tension, and a deeper insight into how this is achieved would require studies involving labeled precursors. As can be seen in Table 3, all four compounds lack leishmanicidal activity (up to 100 μg/mL) on *Leishmania amazonensis* and *L. braziliensis* promastigotes in vitro. Moderate cytotoxicity in murine macrophage cells was noted (IC_50_ values around 70 μg/mL), lower compared with control (miltefosine, 21 μg/mL). 

## 3. Materials and Methods

### 3.1. General

Chemicals and solvents were bought from (Sigma-Aldrich, St. Louis, MO, USA). 1D (^1^H, decoupled ^13^C and DEPT), as well as 2D (COSY, NOESY, ROESY, HMQC and HMBC) NMR experiments, were recorded with a Bruker Advance spectrometer (Bruker Biospin AG, Industriestrasse 26, 8117 Fällanden, Switzerland) operating at 500 MHz for ^1^H and 125 MHz for ^13^C. All NMR experiments were carried out at 22 °C in CDCl_3_, and the solvent signals (at 7.27 and 77.0 ppm, respectively) were used as reference. IR spectra were carried out with an ALPHA FT-IR spectrometer (Bruker Biospin AG, Industriestrasse 26, 8117 Fällanden, Switzerland). Melting point in a STUART melting point apparatus SMP30 (Bibby Scientific Ltd., Chelmsford, Essex, UK). Specific rotation in an ADP 450 series polarimeter apparatus (Bibby Scientific Ltd., Chelmsford, Essex, UK). LC-HRMS data were obtained with a Waters Aquity UPLC and Waters XEVO-G2 (CSH C18, 1.7 μm, 2.1 × 100 mm, Waters Corp, Milford, Worcester County, MA, USA) system. Silica gel 60 (20 mm × 300 mm, 70–230 mesh, Merck, Kenilworth, NJ, USA). Preparative HPLC separations were performed with an Agilent 1200 Infinity series system (Agilent, Santa Clara, CA, USA) equipped with an X-Terra prep RP-18 column (150 mm × 10 mm i.d., 5 μm, Waters, Milford, MA, USA) at 25 °C and with the flow rate at 4.7 mL/min. The HPLC system was equipped with a diode array detector operating at 210 and 230 nm. The recorded spectras and chromatographs are presented in the Supporting Materials.

### 3.2. Plant Material

*Trichilia adolfi* Harms. (Meliaceae) was identified at the National Herbarium of Bolivia (LPB), where a voucher specimen (AS-092) is kept. It was collected in June 2017 (14°21′438″ S, 67°34′728″ W) in the tropical humid forest in the North of La Paz, Bolivia. 

### 3.3. Extraction and Isolation

Milled dried bark (1.0 kg) was macerated with 5.0 L ethanol for 72 h at room temperature to provide a crude extract after evaporation of the solvent (22.0 g, 2.2% of bark weight). This was then re-suspended in aqueous methanol (H_2_O: MeOH, 80:20, 0.5 L) and extracted three times with 0.5 L *n*-heptane, yielding 3.1 g (14% of the crude extract) after evaporation of the solvent. This was followed by the extraction of the aqueous methanol phase with CHCl_3_ (0.5 L), yielding 6.4 g (29% of the crude extract) after evaporation of the solvent, whereafter the aqueous phase was freeze-dried to yield 12.5 g (57% of the crude extract). The CHCl_3_ fraction was subjected to SiO_2_ gel column chromatography (1:30, *w*/*w*) eluted with mixtures of n-heptane: EtOAc (from 8:1 to 2:8 *v*/*v*). TLC analysis provided four main fractions from the least polar to the most polar: F1 (2.5 g), F2 (0.27 g), F3 (0.77 g) and F4 (0.68 g). Further fractionations were performed with fractions F1, F2 and F3 using a semi-preparative HPLC (*vide supra* for details), and eluted with mixtures of solvent A (3 mM formic acid in water) and solvent B (acetonitrile) [20]. The elution was performed in gradient mode, starting with A:B 85:15, reduced linearly to 77:23 in 5 min, a second linear decrease to 74:26 in 25 min, a third linear decrease to 60:40 in 30 min, and finishing at 54:46 for 45 min. All samples were filtered through a 0.25 µm membrane filter prior to HPLC fractionations. Trichilianone C (**3**) was obtained from F1 (30 mg, *t*_R_ = 15.6 min, 0.14% of the raw extract). From F2, crude trichilianone A (**1**) (50 mg) was obtained after SiO_2_ column chromatography eluting with n-heptane: EtOAc 8:2 (*v*/*v*, isocratic). This fraction was further purified by crystallization in 0.5 mL methanol to which 1 mL water was added slowly. Further purification by the HPLC procedure yielded pure **1** (16 mg, *t*_R_ = 10.7 min, 0.070% of the raw extract). Additionally, from F2, trichilianone D (**4**) was isolated following the same procedure (4.9 mg, *t*_R_ = 6.63 min, 0.022%). From F3, trichilianone B (**2**) was isolated after SiO_2_ column chromatography of F3 eluting with n-heptane: EtOAc 6:4 (*v*/*v*, isocratic) to obtain a fraction that yielded pure **2** after HPLC purification (5.0 mg, *t*_R_ = 9.85 min, 0.022% of the raw extract).

### 3.4. Compounds Isolated 

*Trichilianone A* (**1**) was obtained as white crystals: ${\left[\alpha \right]}_{D}^{20}$ = +65° (*c.* 1.00, CHCl_3_). m.p. 181 °C. IR (film) ν_max_ 3017 (OH), 1729 (carboxyl group), 1689 (ketone) cm^−1^; ^1^H NMR (500 MHz, CDCl_3_) and ^13^C NMR (125 MHz, CDCl_3_) data, see Table 1 and Table 2, as well as Supporting Information; LC-HRMS, 543.2252 [M + H]^+^ (calc. for C_29_H_35_O_10_, 543.2230).

*Trichilianone B* (**2**) was obtained as white amorphous powder: ${\left[\alpha \right]}_{D}^{20}$ = + 140° (*c.* 0.50, CHCl_3_). m.p. 145 °C. IR (film) ν_max_ 3471 (OH), 1731 (carboxyl group) cm^−1^; ^1^H NMR (500 MHz, CDCl_3_) and ^13^C NMR (125 MHz, CDCl_3_) data, see Table 1 and Table 2, as well as Supporting Information; LC-HRMS, 545.2395 [M + H]^+^ (calc. for C_29_H_37_O_10_ 545.2387).

*Trichilianone C* (**3**) was obtained as white amorphous powder: ${\left[\alpha \right]}_{D}^{20}$ = + 45° (c. 1.00, CHCl_3_). m.p. 151 °C. IR (film) ν_max_ 3012 (C-H stretching), 1733 (carboxyl group), 755 (C-H bending) cm^−1^; ^1^H NMR (500 MHz, CDCl_3_) and ^13^C NMR (125 MHz, CDCl_3_) data, see Table 1 and Table 2, as well as Supporting Information; LC-HRMS, 615.2821 [M + H]^+^ (calc. for C_33_H_43_O_11_, 615.2805).

*Trichilianone D* (**4**) was obtained as white amorphous powder: ${\left[\alpha \right]}_{D}^{20}$ = + 55 (*c.* 1.00, CHCl_3_). m.p. 175 °C. IR (film) ν_max_ 2970 (OH), 1732 (carboxyl group), 1687 (ketone) cm^−1^; ^1^H NMR (500 MHz, CDCl_3_) and ^13^C NMR (125 MHz, CDCl_3_) data, see Table 1 and Table 2, as well as Supporting Information; LC-HRMS, 559.2171 [M + H]^+^ (calc. for C_29_H_35_O_11_, 559.2179).

### 3.5. In Vitro Leishmanicidal Activity

Leishmanicidal activity was assayed according to Williams [21], with some modification. Promastigotes of Leishmania: *L. amazonensis*, Clone 1, NHOM-BR-76-LTB-012 (Lma, donated by the Paul Sabatier Université, France) and *L. braziliensis* M2904 C192 RJA (M2904, donated by Dr. Jorge Arévalo from Universidad Peruana Cayetano Heredia, Peru). The strains were cultured in Schneider’s insect medium (pH 6.2), supplemented with 10% FBS and incubated in 96-microwell plates at 26 °C. Briefly, promastigotes in logarithmic phase of growth, at concentration of 106 parasites/mL, were exposed to samples dissolved in DMSO (1%) at different concentrations (3.1–100 μg/mL). Miltefosine (3.1–100 μg/mL, IDPS, France) was used as control drug. The microwell plates were incubated for 72 h at 26 °C, the optical density of each well was measured and the IC_50_ values calculated. All assays were performed in triplicate.

### 3.6. Cytotoxicity

The Raw 264.7 murine macrophage cell line was purchased from the American-Type Culture Collection (ATCC-TIB71). The cells were maintained in DMEM-HG medium supplemented with 10% fetal bovine serum, 100 U/mL of penicillin and 100 μg/mL of streptomycin, and sodium bicarbonate (2.2 g/L) in humidified atmosphere at 37 °C with 5% CO_2_. Samples were prepared as described above and added (in 100 μL DMSO) at different concentrations (6.2–200 μg/mL). Medium blank, control drugs and cell growth controls were included to evaluate cell viability. The plates were incubated for 72 h at 37 °C with 5% CO_2_. After incubation for the indicated time, the cells were washed, after which 10 μL of Resazurin reagent (2.0 mM) was added. They were further incubated at 37 °C for 3 h in a humidified incubator. The IC_50_ values were assessed using a fluorometric reader (540 nm excitation, 590 nm emission) and Gen5 software. All assays were performed in triplicate.

## 4. Conclusions

The present study was initiated by the leishmanicidal activity observed in the extract of the bark of *Trichilia adolfi*, but came to focus on the characterization of limonoid-type metabolites. The four new compounds trichilinones A-D (**1**–**4**), with five fused rings and related to the hortiolide-type limonoids, were isolated from the extract of the bark, and their structures were elucidated after an extensive spectroscopic analysis. This was essentially based on the elemental composition determined by high-resolution mass spectrometry, and 1D NMR as well as 2D NMR experiments. The cytotoxicity of the pure compounds was evaluated in vitro in cell cultures of murine macrophages (Raw 264.7), while the leishmanicidal properties were evaluated in the two leishmania-promastigote strains *Leishmania amazoniensis* and *L. braziliensis*. While all four compounds were found to be weakly cytotoxic, they lacked any leishmanicidal activity. It must therefore be assumed that other metabolites of the bark of *Trichilia adolfi* are responsible for this activity, or that it is due to a synergistic effect of the existing metabolites in the crude extract.

## Figures and Tables

**Table 1:** ^1^H NMR spectroscopic data for **1**–**4** recorded in CDCl_3_. The chemical shifts are in ppm, multiplicities and coupling constants (in Hz) are given in parentheses. The solvent signal at 7.27 ppm was used as a reference.

H	1	2	3	4
1	-	4.42 (dd, 12.3, 3.9)	4.42 (dd, 12.1, 4.1)	-
2a	5.50 (s)	3.33 (dd, 14.1, 12.3)	3.27 (dd, 14.0, 12.1)	5.50 (s)
2b	-	2.87 (dd, 14.1, 3.9)	2.91 (dd, 14.0, 4.1)	-
5	2.62 (dd, 11.7, 1.5)	3.01 (dd, 12.9, 2.3)	3.09 (dd, 12.6, 2.3)	2.60 (dd, 11.6, 1.6)
6a	3.79 (dd, 16.8, 1.5)	3.07 (dd, 15.0, 2.3)	2.93 (dd, 14.2, 2.3)	3.82 (dd, 18.6, 1.6)
6b	2.72 (dd, 16.8, 11.7)	2.69 (dd, 15.0, 12.9)	2.66 (dd, 14.2, 12.6)	2.78 (dd, 18.6, 11.6)
11	4.34 (dd, 2.6, 1.9)	4.33 (dd, 2.3, 1.6)	5.49 (d, 1.7)	4.34 (dd, 2.2, 2.2)
12	4.91 (d, 1.9)	4.92 (d, 1.6)	5.36 (d, 1.7)	5.62 (d, 2.2)
16a	3.06 (dd, 18.8, 7.9)	3.02 (dd, 18.5, 6.6)	3.02 (dd, 18.5, 7.5)	3.11 (dd, 18.5, 7.9)
16b	2.69 (dd, 12.5, 18.8)	2.72 (dd, 18.5, 12.4)	2.73 (dd, 18.5, 13.0)	2.76 (dd, 18.5, 12.1)
17	3.37 (dd, 12.5, 7.9)	3.35 (dd, 12.4, 6.6)	3.30 (dd, 13.0, 7.5)	3.36 (dd, 12.1, 7.9)
18	0.95 (s)	0.90 (s)	0.77 (s)	0.97 (s)
19	1.67 (s)	1.55 (s)	1.31 (s)	1.69 (s)
21	7.33 (dd, 1.6, 0.9)	7.34 (dd, 1.7, 0.9)	7.32 (dd, 1.7, 1.0)	7.32 (dd, 1.7, 1.7)
22	6.30 (dd, 1.8, 0.9)	6.30 (dd, 1.7, 0.9)	6.43 (dd, 1.7, 1.0)	4.93 (t, 1.7)
23	7.46 (dd, 1.6, 1.8)	7.45 (dd, 1.7, 1.7)	7.43 (dd, 1.7, 1.7)	-
24	1.47 (s)	1.34 (s)	1.37 (s)	1.46 (s)
25	1.38 (s)	1.49 (s)	1.46 (s)	1.40 (s)
26	1.78 (s)	1.67 (s)	1.84 (s)	1.78 (s)
2′	2.13 (s)	2.11 (s)	2.07 (s)	2.14 (s)
MeO	3.67 (s)	3.68 (s)	3.73 (s)	3.69 (s)
2”	-	-	2.66 (dd, 7.1, 6.9)	-
3”	-	-	1.26 (d, 6.9)	-
4”	-	-	1.32 (d, 7.1)	-
OH	3.66 (d, 2.6)	not observed	-	3.76 (d, 2.0)

**Table 2:** ^13^C NMR spectroscopic data for **1**–**4** recorded in CDCl_3_. The chemical shifts are in ppm, the multiplicities were determined by DEPT and HMQC experiments. The solvent signal at 77.0 ppm was used as a reference.

C	1	2	3	4
1	180.2, s	90.8, d	90.6, d	180.4, s
2	94.2, d	37.3, t	37.2, t	94.4, d
3	168.7, s	170.9, s	170.3, s	168.6, s
4	83.1, s	84.9, s	84.4, s	83.1, s
5	49.7, d	43.2, d	42.8, d	49.8, d
6	35.0, t	37.7, t	37.5, t	35.2, t
7	174.4, s	173.8, s	172.9, s	174.7, s
8	77.5, s	75.5, s	75.9, s	77.6, s
9	57.8, s	62.8, s	60.9, s	58.0, s
10	56.1, s	57.2, s	56.8, s	56.2, s
11	82.8, d	83.6, d	83.1, d	82.6, d
12	91.3, d	91.2, d	85.2, d	91.6, d
13	54.3, s	54.2, s	55.1, s	53.7, s
14	59.2, s	62.8, s	63.1, s	58.9, s
15	209.6, s	211.8, s	211.0, s	208.5, s
16	44.2, t	44.8, t	44.5, t	43.3, t
17	41.8, d	42.2, d	41.8, d	42.3, d
18	12.8, q	13.1, q	12.7, q	13.4, q
19	19.5, q	22.6, q	21.9, q	19.3, q
20	120.6, s	121.2, s	121.0, s	132.0, s
21	140.3, d	140.2, d	140.4, d	173.3, s
22	109.9, d	109.9, d	110.1, d	148.7, d
23	143.7, d	143.7, d	143.7, d	70.6, t
24	31.0, q	30.9, q	30.9, q	31.2, q
25	28.6, q	27.0, q	27.0, q	28.6, q
26	14.4, q	15.2, q	15.2, q	14.6, q
1′	172.0, s	171.9, s	168.5, s	171.9, s
2′	20.9, q	20.9, q	20.7, q	20.8, q
OMe	51.9, q	51.9, q	52.2, q	52.0, q
1”	-	-	175.0, s	-
2”	-	-	34.2, d	-
3”	-	-	18.3, q	-
4”	-	-	19.3, q	-

**Table 3:** Leishmanicidal activity against *leishmanial* promastigotes (*L.a*: *Leishmania amazonensis*; *L.b.*: *L. braziliensis*) and cytotoxicity in Raw murine macrophage cell cultures (Raw), of compounds **1**–**4** and the positive control miltefosine. The data are given as IC_50_ values in μg/mL (see Experimental section for details).

Compound	*L.a.*	*L.b*	Raw
**1**	>100	>100	64 ± 23
**2**	>100	>100	75 ±18
**3**	>100	>100	70 ± 27
**4**	>100	>100	76 ± 23
Miltefosine	5.0 ± 0.2	4.1 ± 0.5	21 ± 2.0

## Data Availability

All data are available in this publication and in the Supplementary Materials.

## Supplementary Materials

The following are available online. They contain 1D and 2D NMR spectra of all isolated compounds.

## References

[1] Ulloa C., Acevedo-Rodríguez P., Beck S., Belgrano M.J., Bernal R., Berry P.E., Brako L., Celis M., Davidse G., Forzza R.C. (2018). Vascular Plants of the Americas (VPA) Website. Tropicos, Botanical Information System at the Missouri Botanical Garden, St. Louis, Missouri, USA. http://legacy.tropicos.org/Project/VPA.

[2] Queveno C., Bourdy G., Gimenez A. (1999). Tacana.

[3] Bourdy G., DeWalt S.J., Chavez de Michel L.R., Roca A., Deharo E., Muñoz V., Balderrama L., Quenevo C., Gimenez A. (2000). Medicinal plants uses of the Tacana, an amazonian Bolivian ethnic group. J. Ethnopharmacol..

[4] Arévalo-Lopéz D., Nina N., Ticona J.C., Limachi I., Salamanca E., Udaeta E., Paredes C., Espinoza B., Serato A., Garnica D. (2018). Leishmanicidal and cytotoxic activity of plants used in Tacana traditional medicine (Bolivia). J. Etnopharmacol..

[5] Tan Q.G., Luo X.D. (2011). Meliaceous limonoids: Chemistry and biological activities. Chem. Rev..

[6] Nakatani M., James J.C., Nakanishi K. (1981). Isolation and structures of trichilins, anti-feedants against the Southern army worm. J. Am. Chem. Soc..

[7] Cao D.H., Liao S.G., Yang L., Li X.N., Wu B., Zhang P., Guo J., Xiao C.F., Hu H.B., You-Kai Xu Y.K. (2017). Trichiliasinenoids A-C, three 6,7-secomexicanolide limonoids with a 7,29-linkage from *Trichilia sinensis*. Tetrahedron.

[8] Zhang Q., Di Y.T., He H.P., Fang X., Chen D.L., Yan X.H., Zhu F., Yang T.Q., Liu L.L., Hao X.J. (2011). Phragmalin- and mexicanolide-type limonoids from the leaves of *Trichilia connaroides*. J. Nat. Prod..

[9] Rodríguez-Hahn L., Cárdenas J., Arenas C. (1996). Trichavensin, a prieurianin derivative from *Trichilia havanensis*. Phytochemistry.

[10] Morgan E.D. (2009). Azadirachtin, a scientific gold mine. Bioorg. Med. Chem..

[11] Cao D.H., Sun P., Liao S.G., Gan L.S., Yang L., Yao J.N., Zhang Z.Y., Li J.F., Zheng X.L., Xiao Y.D. (2019). Chemical constituents from the twigs and leaves of *Trichilia sinensis* and their biological activities. Phytochem. Lett..

[12] Ji K.L., Zhang P., Li X.N., Guo J., Hu H.B., Xiao C.F., Xie X.Q., Xu Y.K. (2015). Cytotoxic limonoids from *Trichilia americana* leaves. Phytochemistry.

[13] Venkata K.C.N., Rathinavelu A., Bishayee A. (2018). Limonoids: Structure–activity relationship studies and anticancer properties. Stud. Nat. Prod. Chem..

[14] Suarez L.E., Menichini F., Monache F.D. (2002). Tetranortriterpenoids and dihydrocinnamic acid derivatives from *Hortia colombiana*. J. Braz. Chem. Soc..

[15] Liu J.Q., Wang C.F., Li Y., Chen J.C., Zhou L., Qiu M.H. (2012). Limonoids from the leaves of *Toona ciliata* var. yunnanensis. Phytochemistry.

[16] Chan W.R., Gibbs J.A., Taylor D.R. (1967). The limonoids of *Trichilia havanensis* Jacq.: An epoxide rearrangement. Chem. Commun..

[17] Severino V.G.P., Braga P.A.C., da Silva M.F., Fernandes J.B., Vieira P.C., Theodoro J.E., Ellena J.A. (2012). Cyclopropane- and spirolimonoids and related compounds from *Hortia oreadica*. Phytochemistry.

[18] Zhao M., Bai L., Wang L., Toki A., Hasegawa T., Kikuchi M., Abe M., Sakai J.I., Hasegawa R., Bai Y. (2007). Bioactive cardenolides from the stems and twigs of *Nerium oleander*. J. Nat. Prod..

[19] Banthorp D.B. (1970). Terpene biosynthesis. Part 11. Biosynthesis of thujane derivatives in thuja, tanacetum, and juniperus species. Chem. Soc..

[20] Vikram A., Jayaprakasha G.K., Patil B.S. (2007). Simultaneous determination of citrus limonoid aglycones and glucosides by high performance liquid chromatography. Anal. Chim. Acta.

[21] Williams C., Espinosa O.A., Montenegro H., Cubilla L., Capson T.L., Ortega-Barría E., Romero L.I. (2003). Hydrosoluble formazan XTT: Its application to natural products drug discovery for Leishmania. J. Microbiol. Methods.

