# Supplementary material for: Trichilianones A-D, Novel Cyclopropane-Type Limonoids from Trichilia adolfi
Source: Molecules. 2021 Feb 15;26(4):1019. doi: 10.3390/molecules26041019 (PMC7919047; doi:10.3390/molecules26041019)

Figure S1: $^1$H NMR of compound 1 ($CDCl_3$)

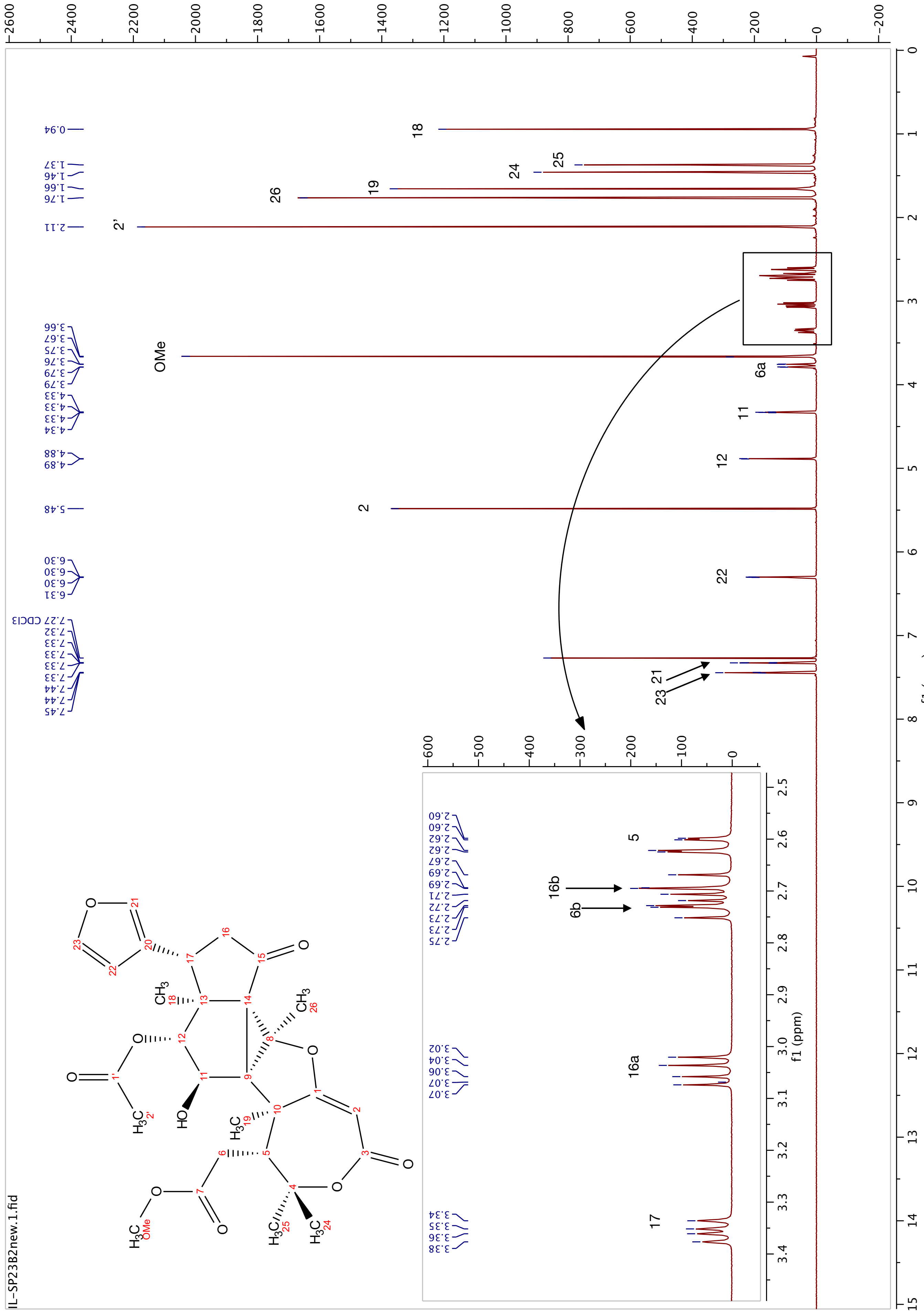

Figure S2: $^{13}C$ NMR of compound 1 ($CDCl_3$)

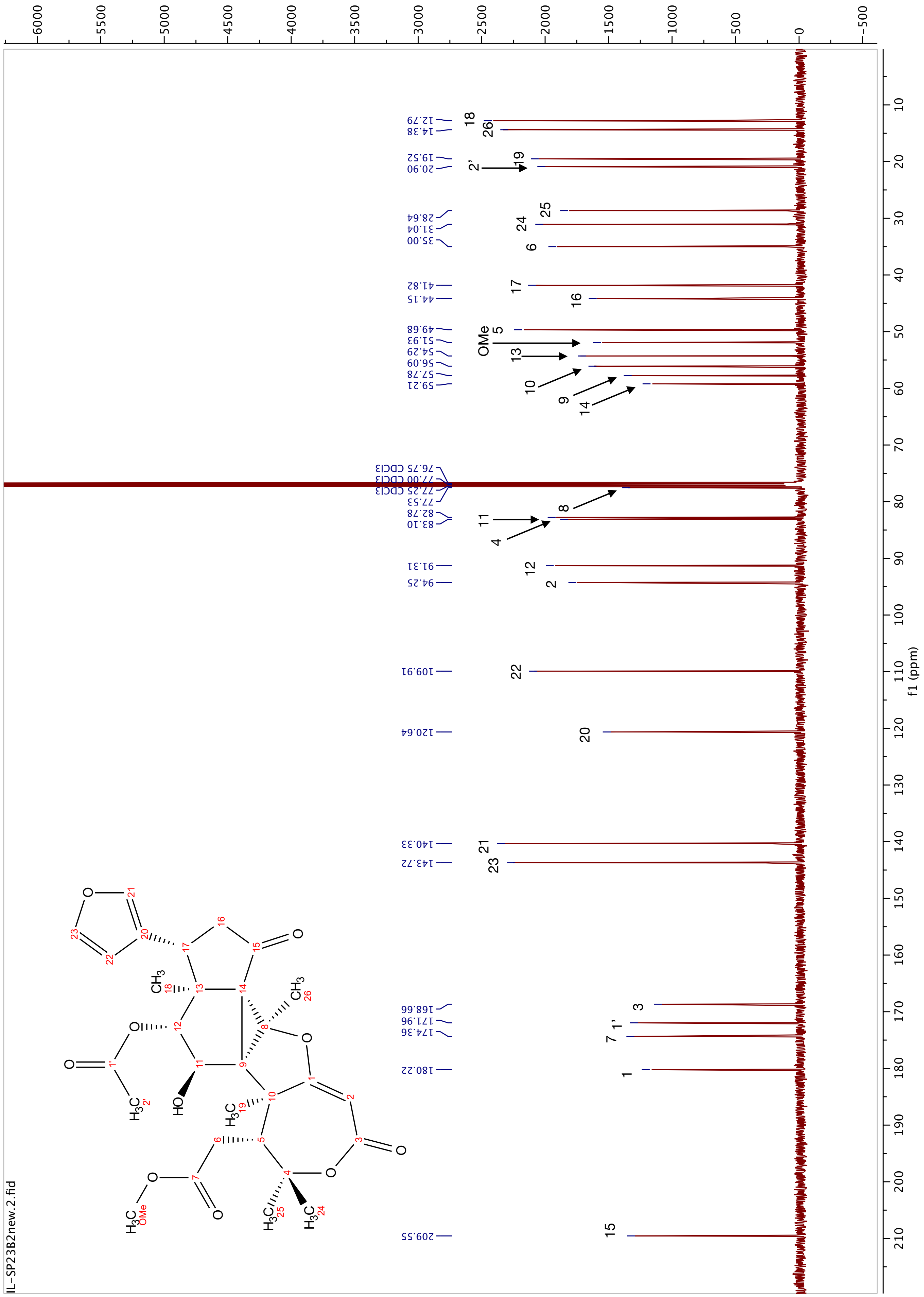

Figure S3: Comparative $^{13}C$ NMR with DEPT135 experiment ($CDCl_3$)

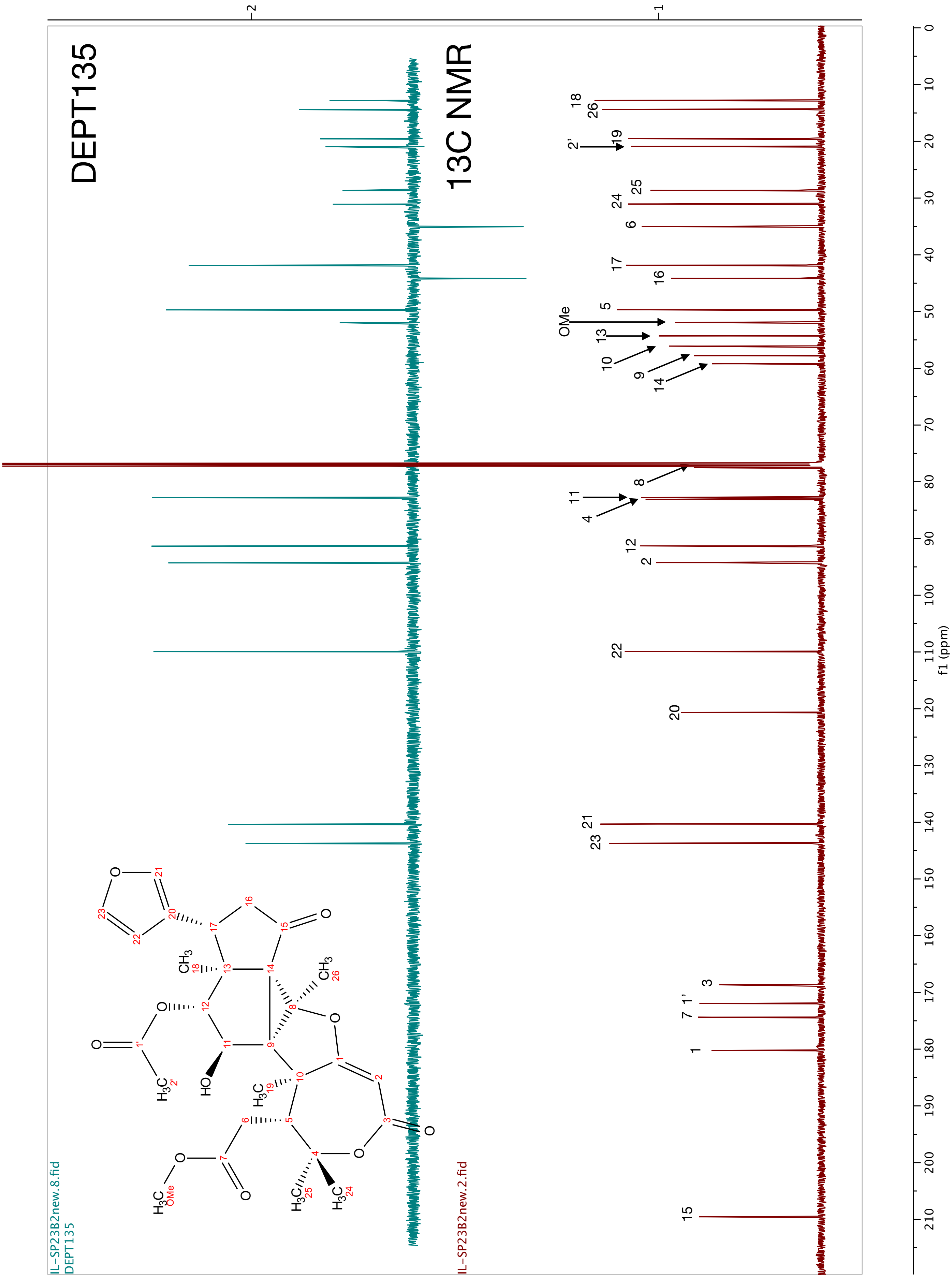

Figure S4: COSY experiment of compound 1 ($CDCl_3$)

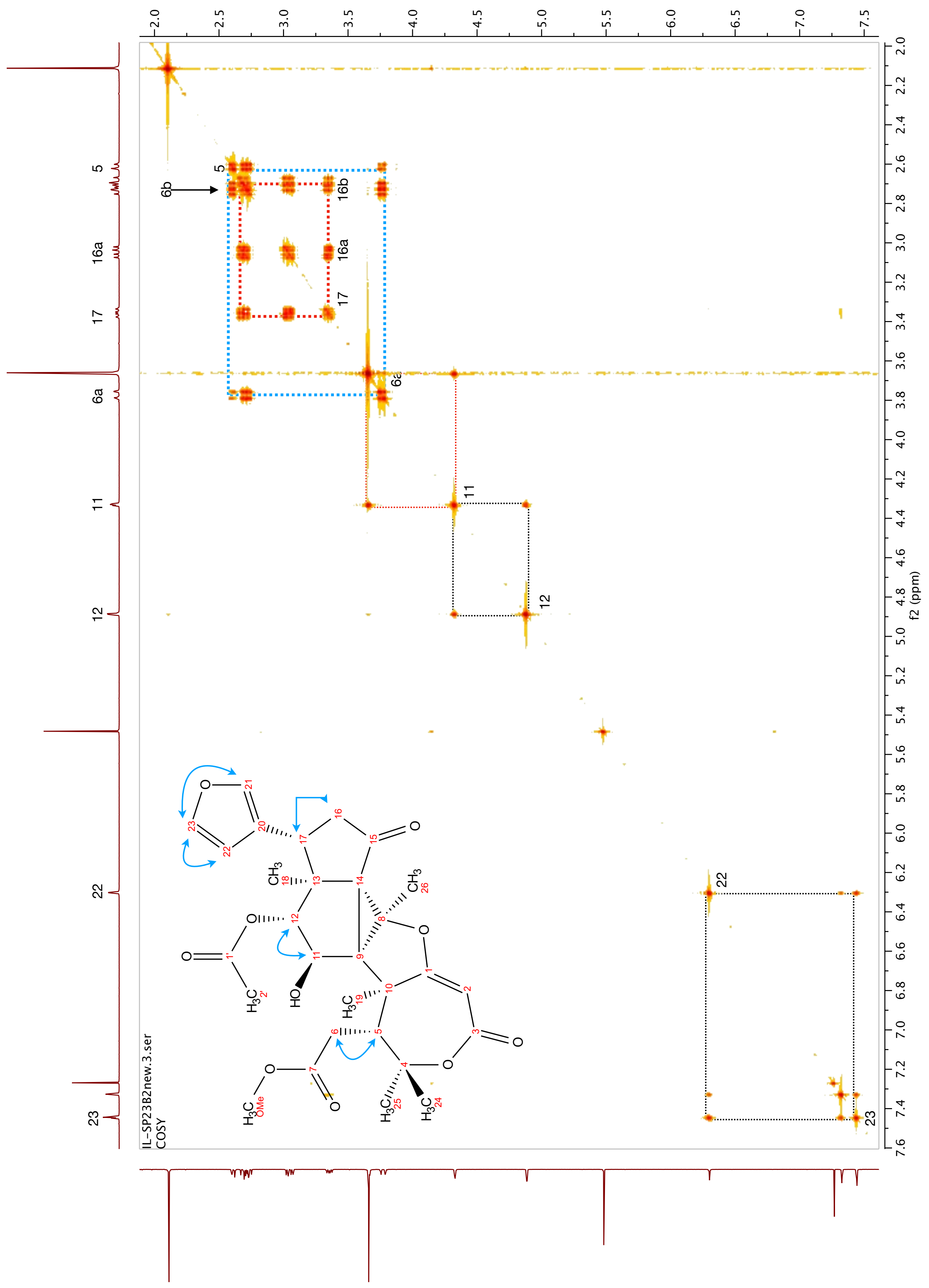

Figure S5: HMQC experiment of compound 1 (CDCl3)

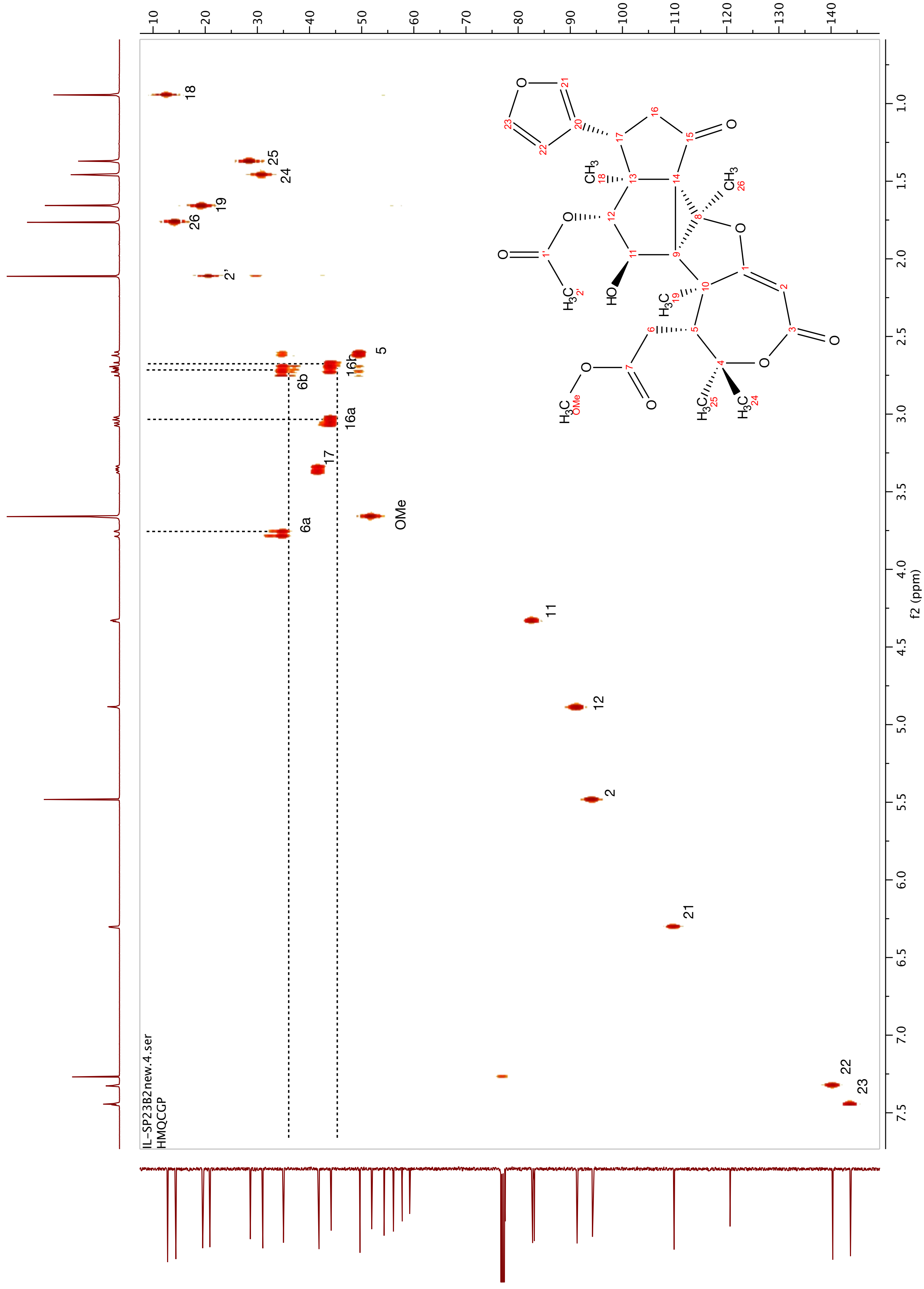

# Figure S6: HMBC experiment of compound 1 ($CDCl_3$)

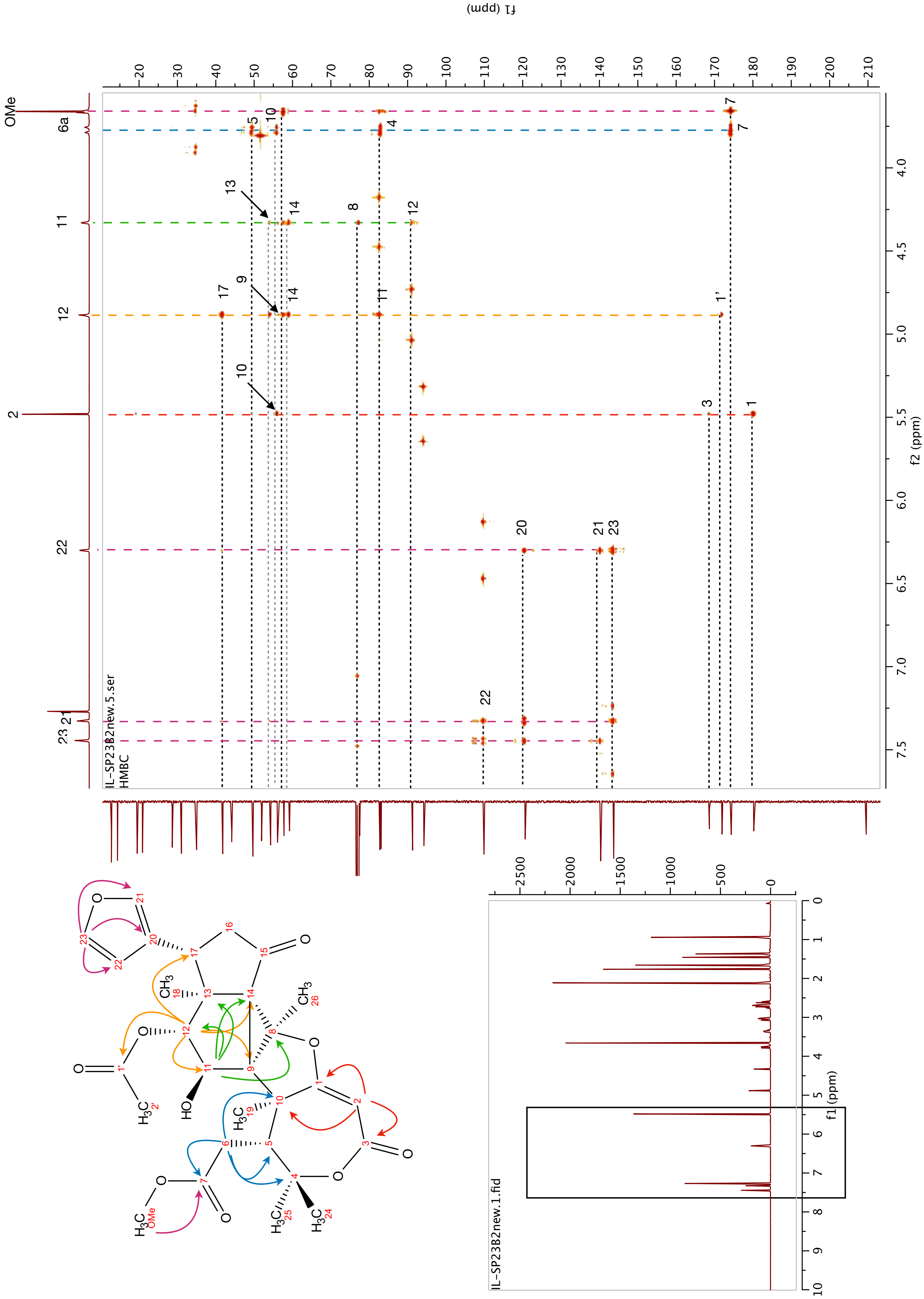

# Figure S7: HMBC experiment of compound 1 ($CDCl_3$)

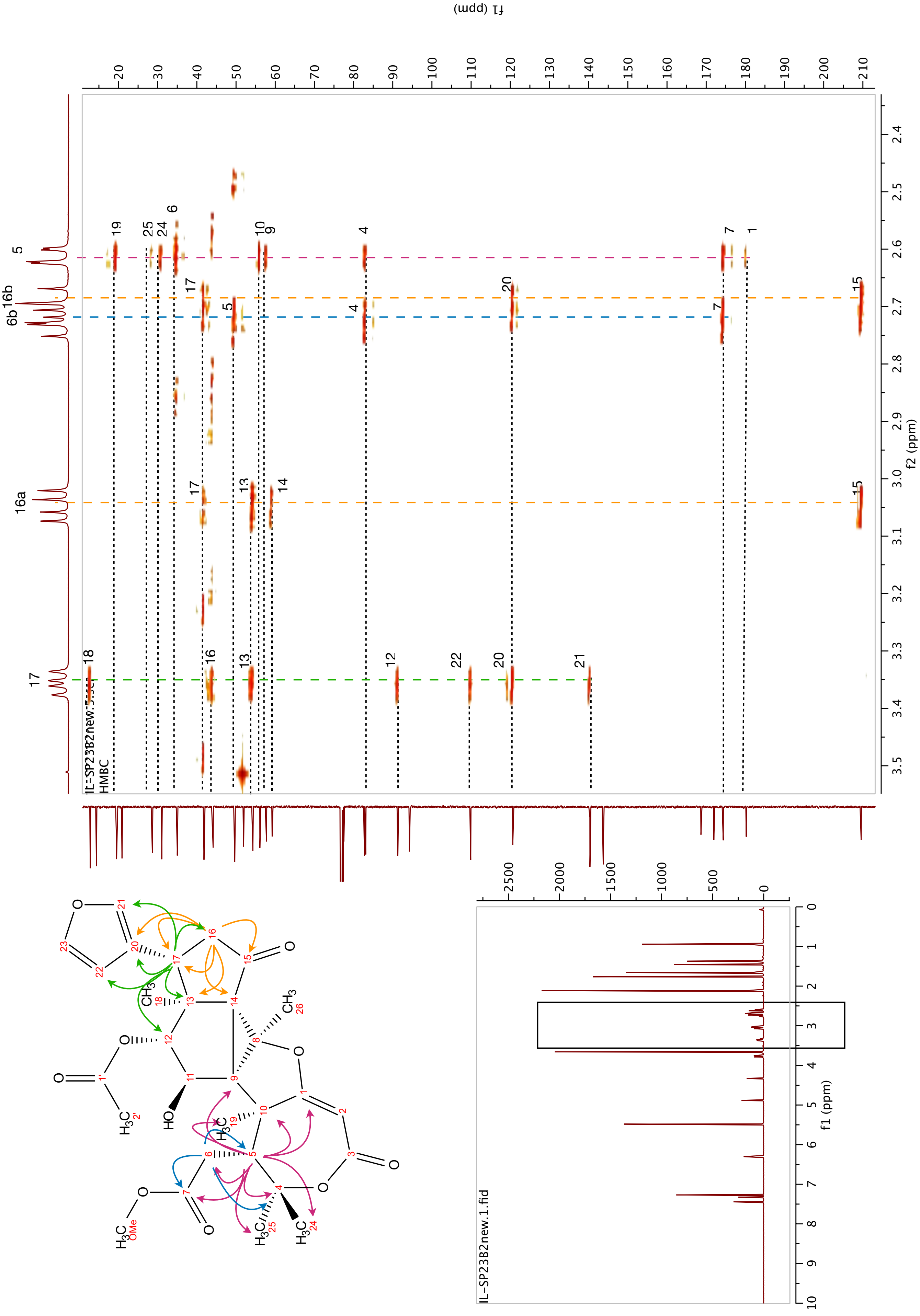

Figure S8: HMBC experiment of compound 1 ($CDCl_3$)

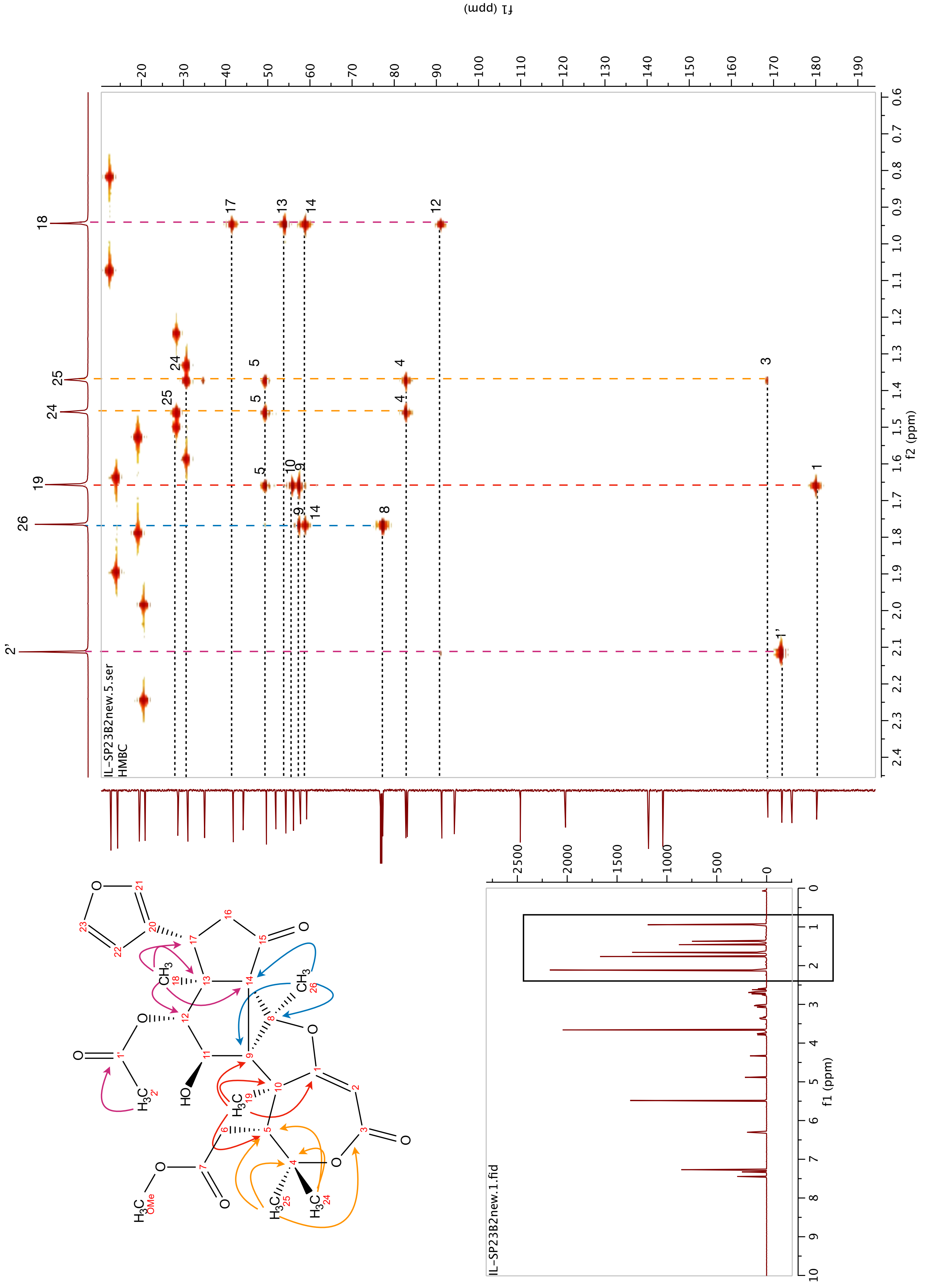

# Figure S9: NOESY experiment of compound 1 ($CDCl_3$)

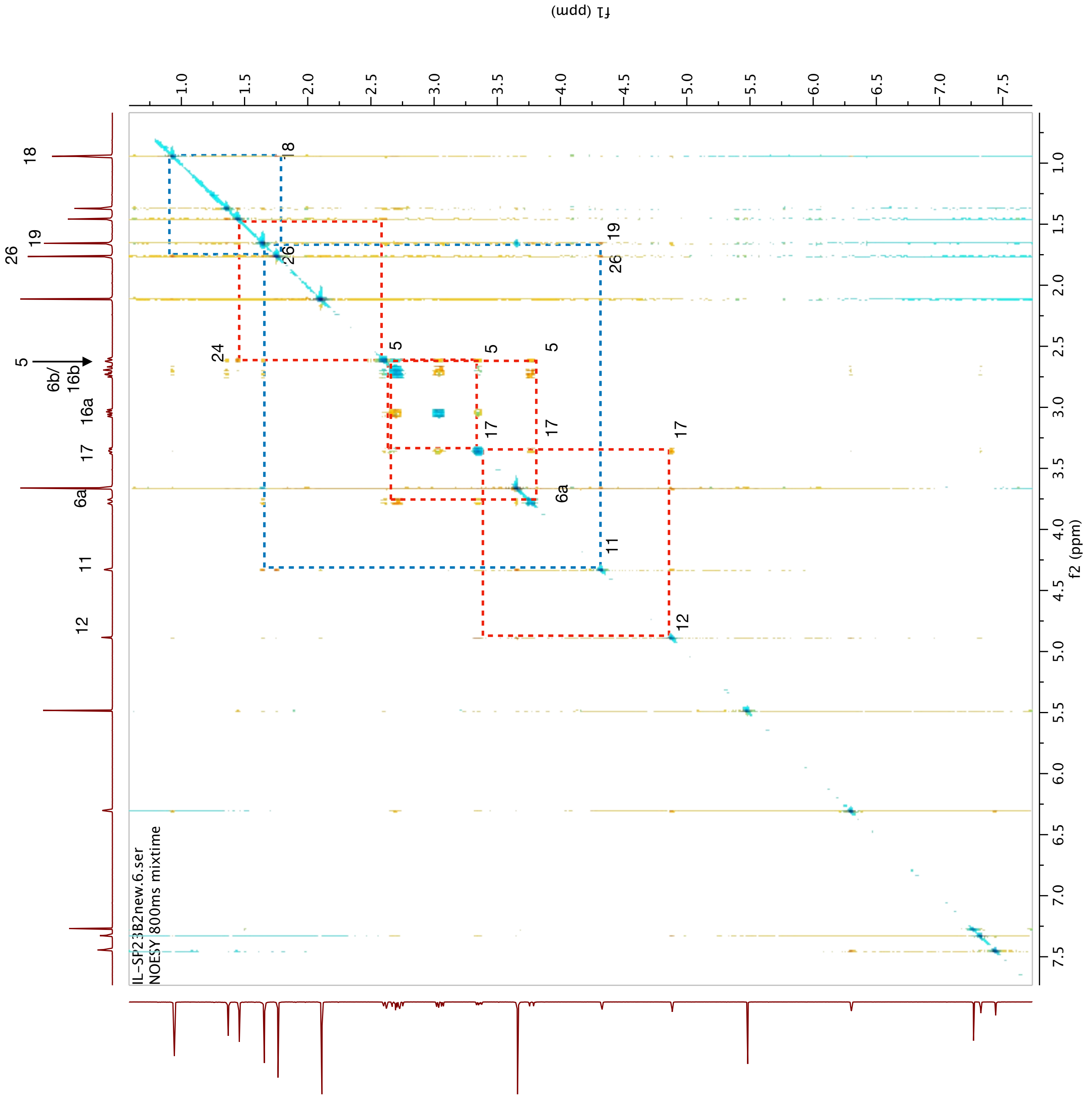


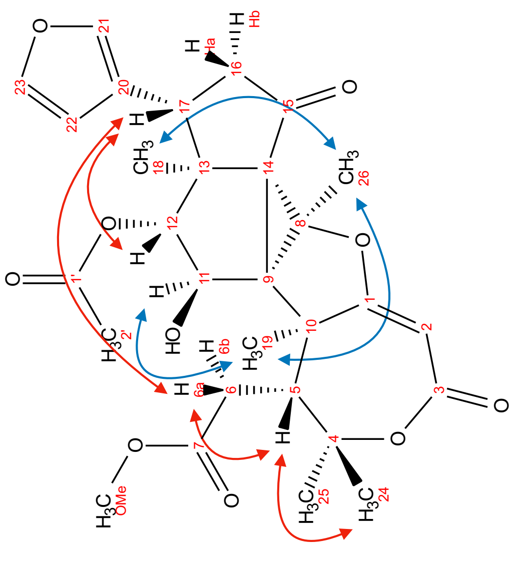

# Figure S10: HRMS and IR spectra of compound 1

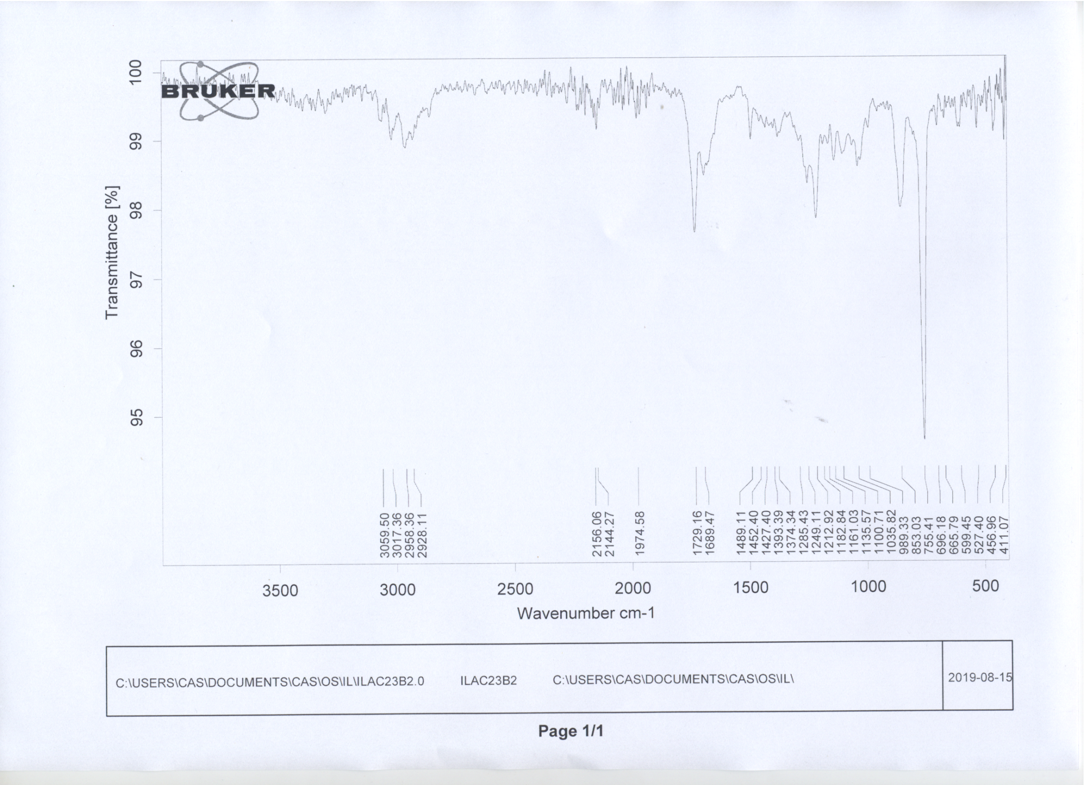


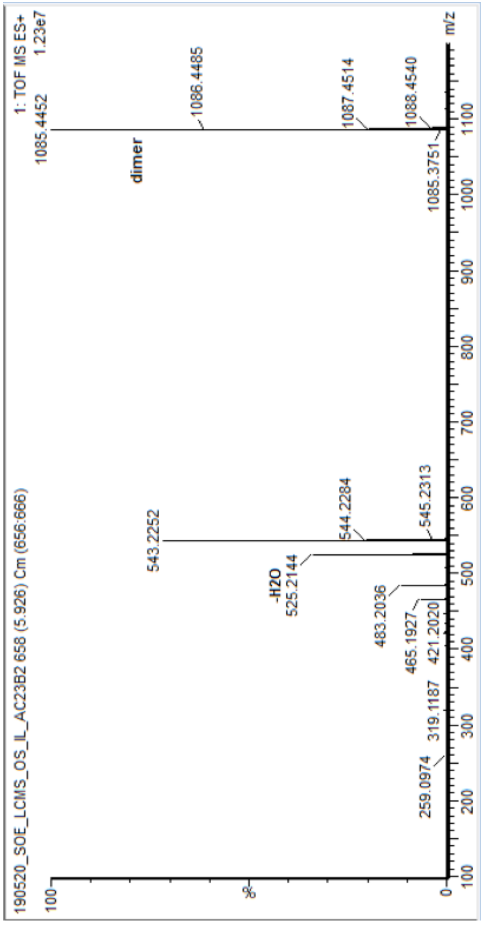


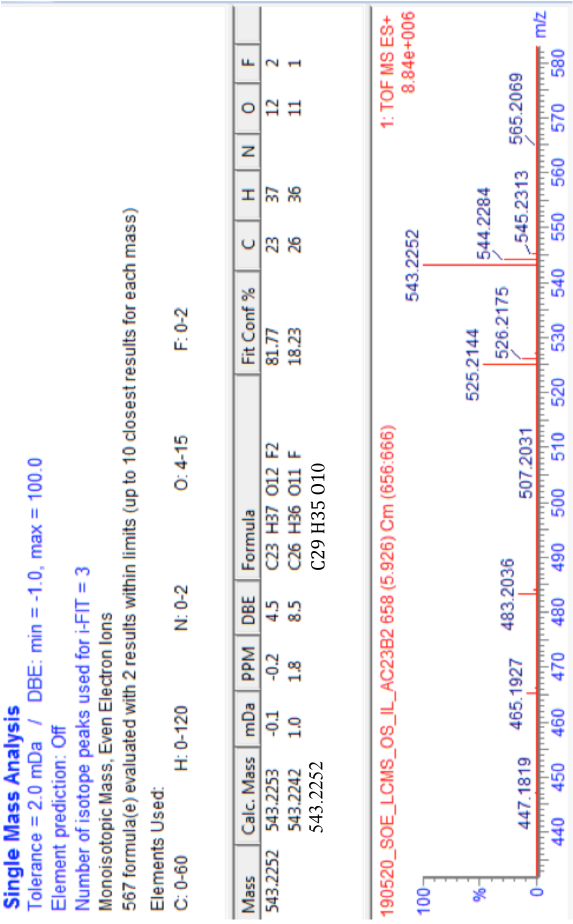

Figure S11: $^{1}H$ NMR of compound 2 ($CDCl_3$)

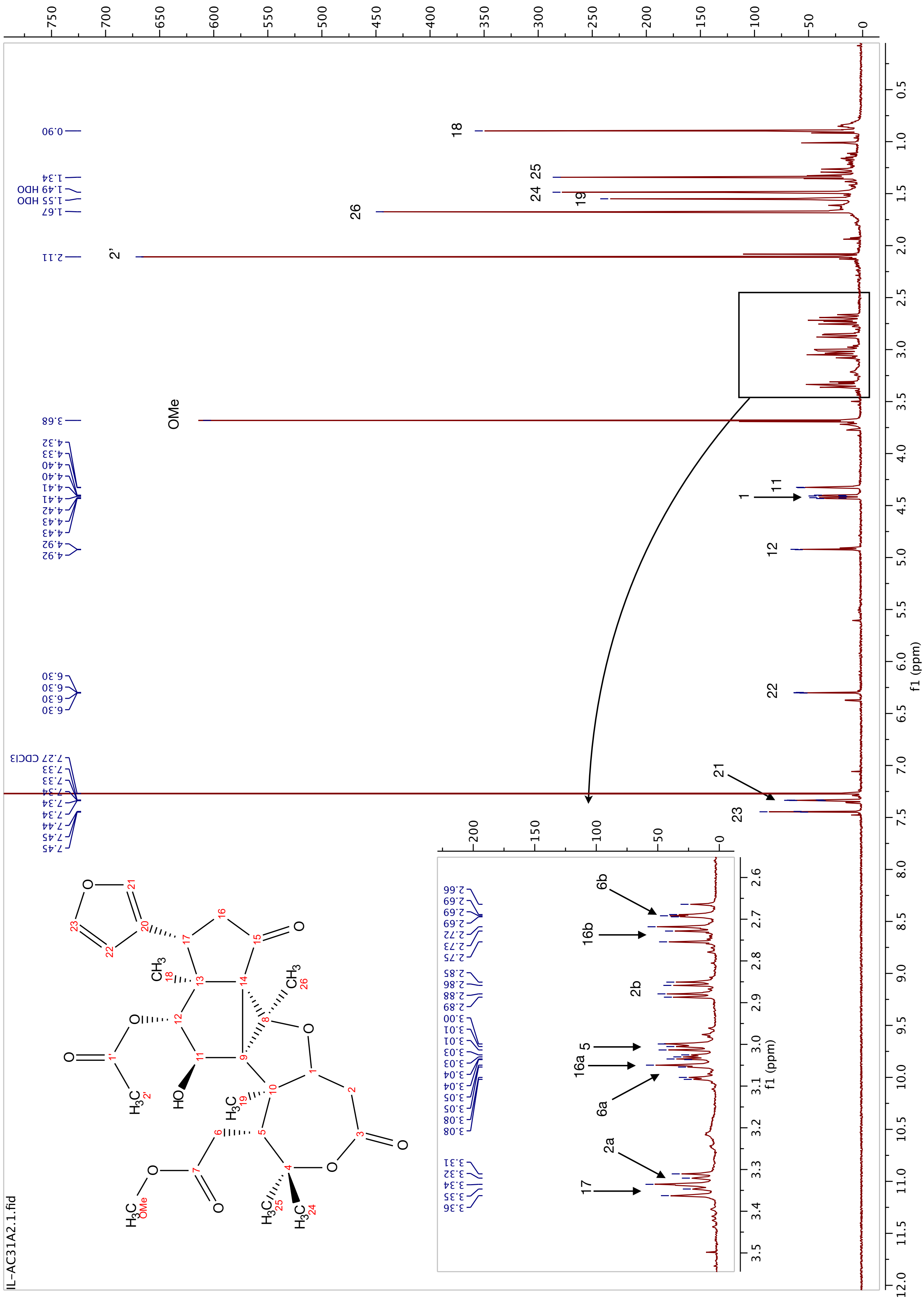

# Figure S12: $^{13}C$ NMR of compound 2 ($CDCl_3$)

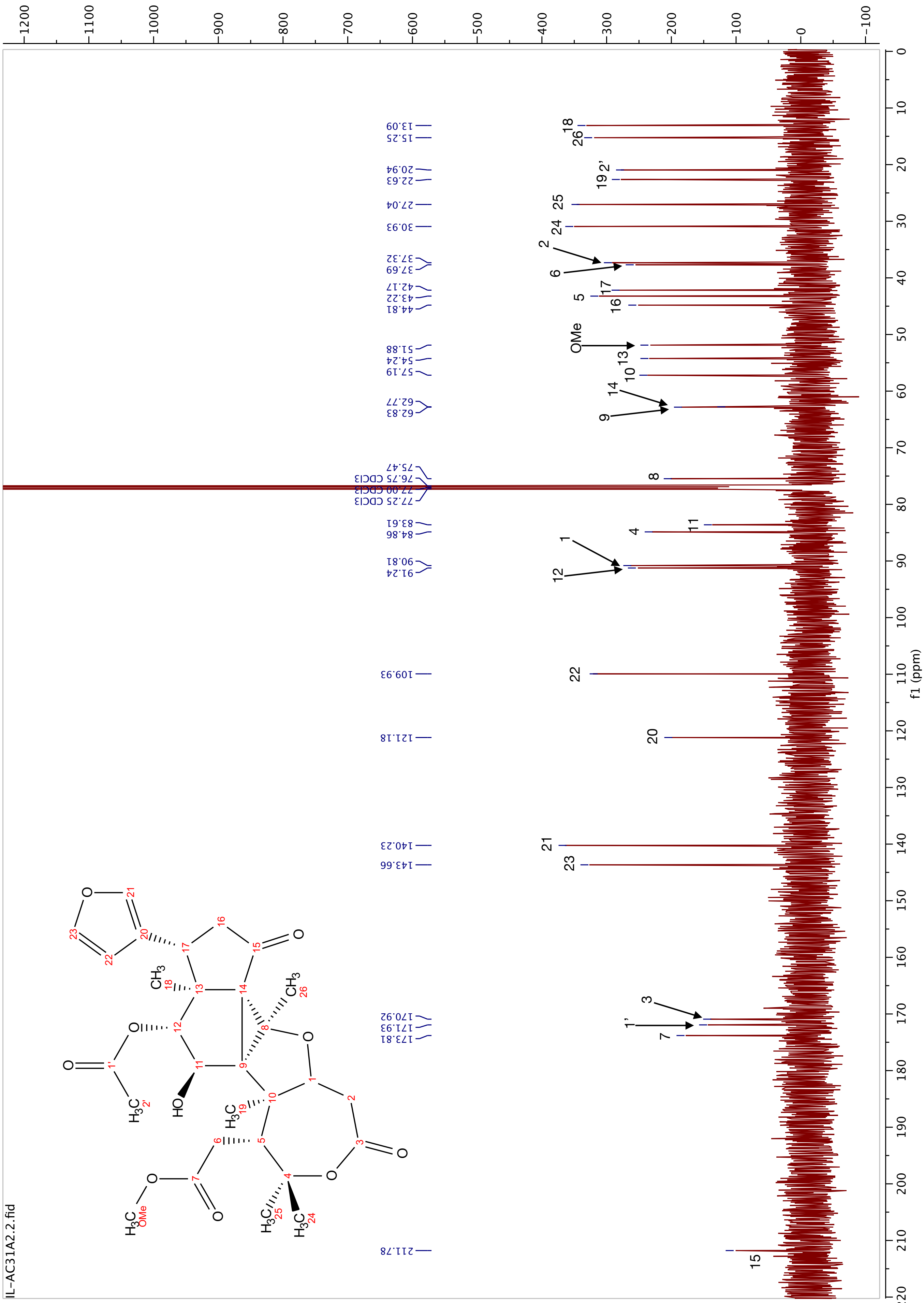

# Figure S13: COSY experiment of compound 2 ($CDCl_3$)

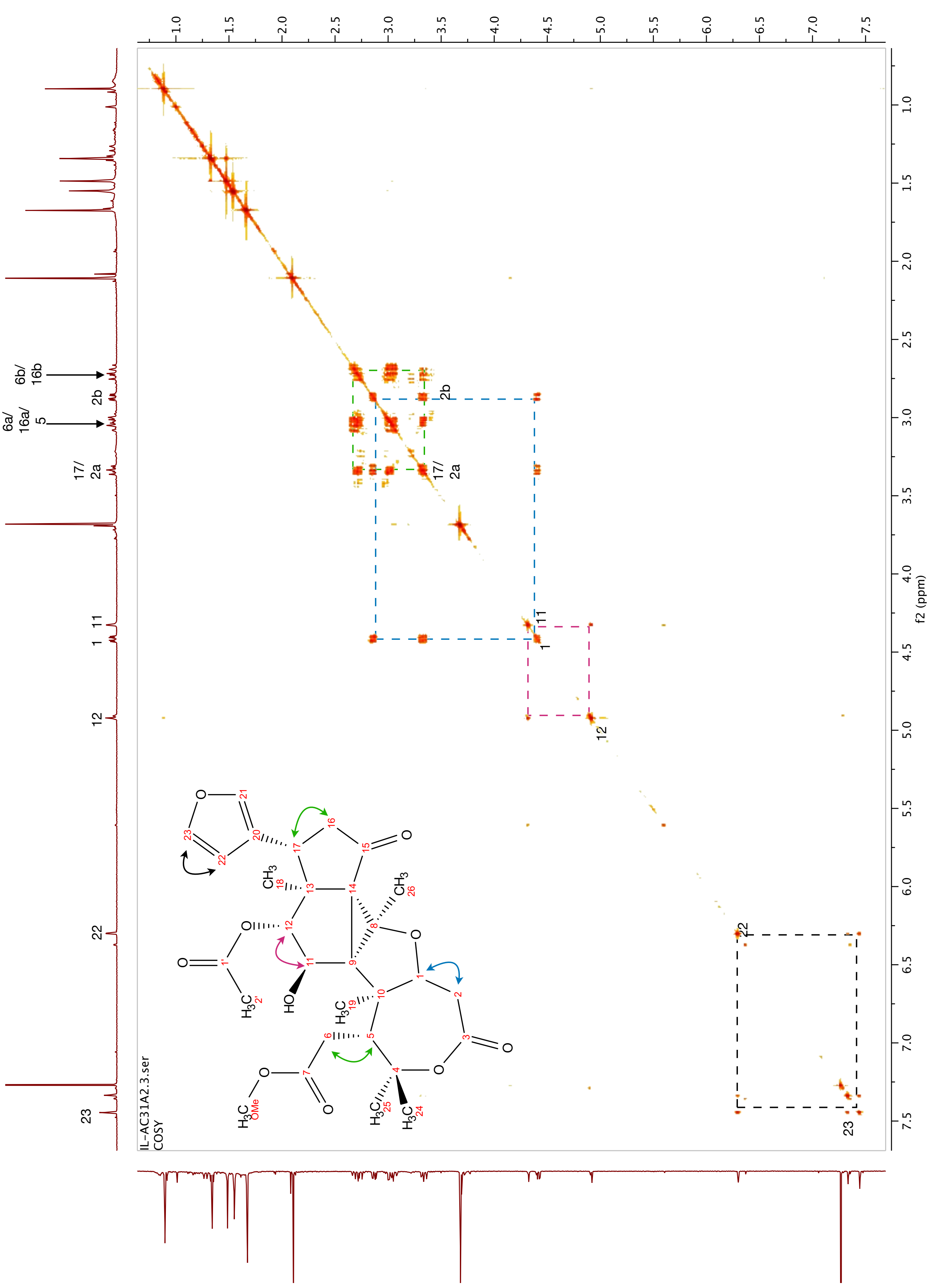

# Figure S14: HMQC experiment of compound 2 (CDCl3)

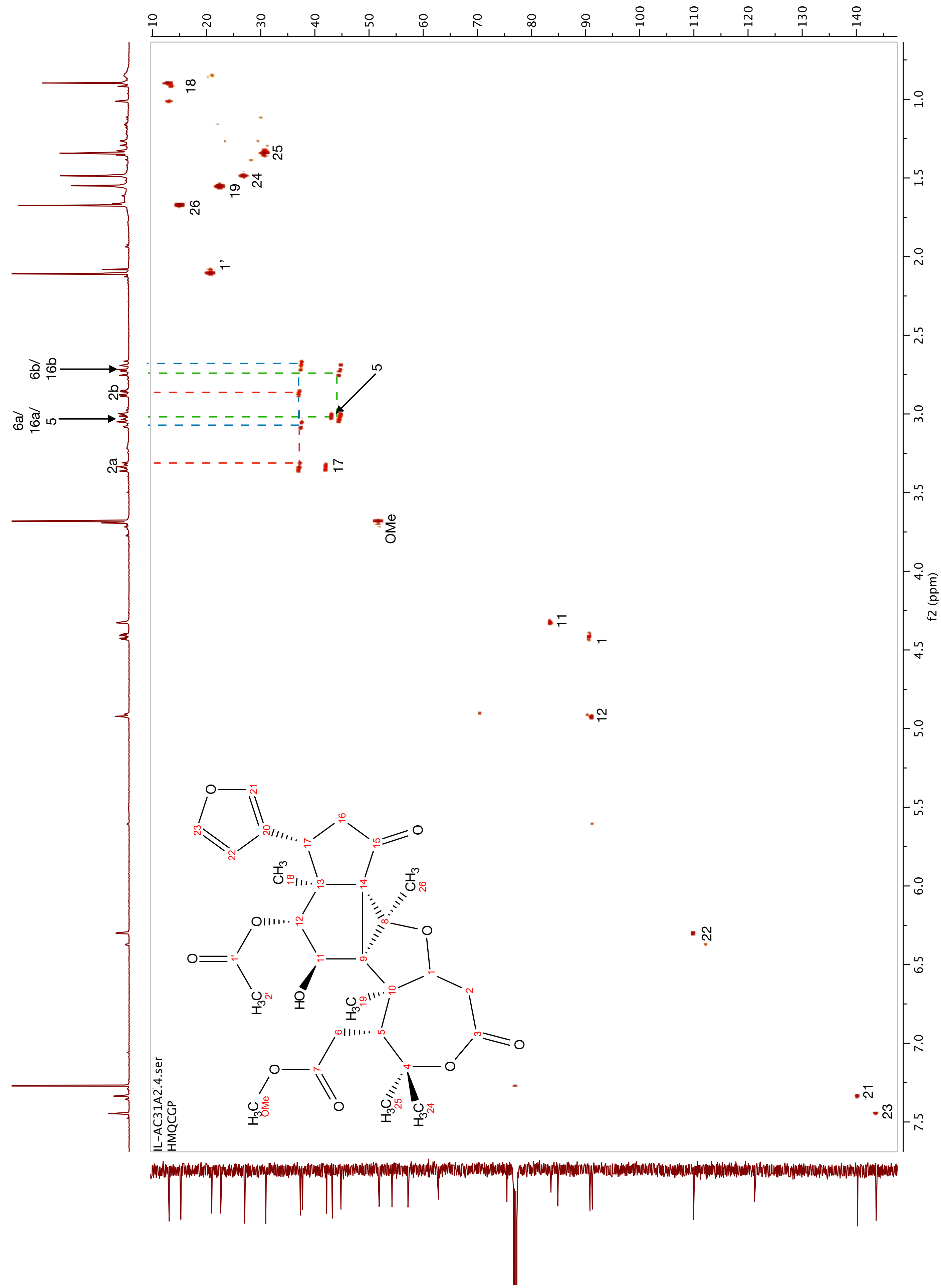

# Figure S15: HMBC experiment of compound 2 ($CDCl_3$)

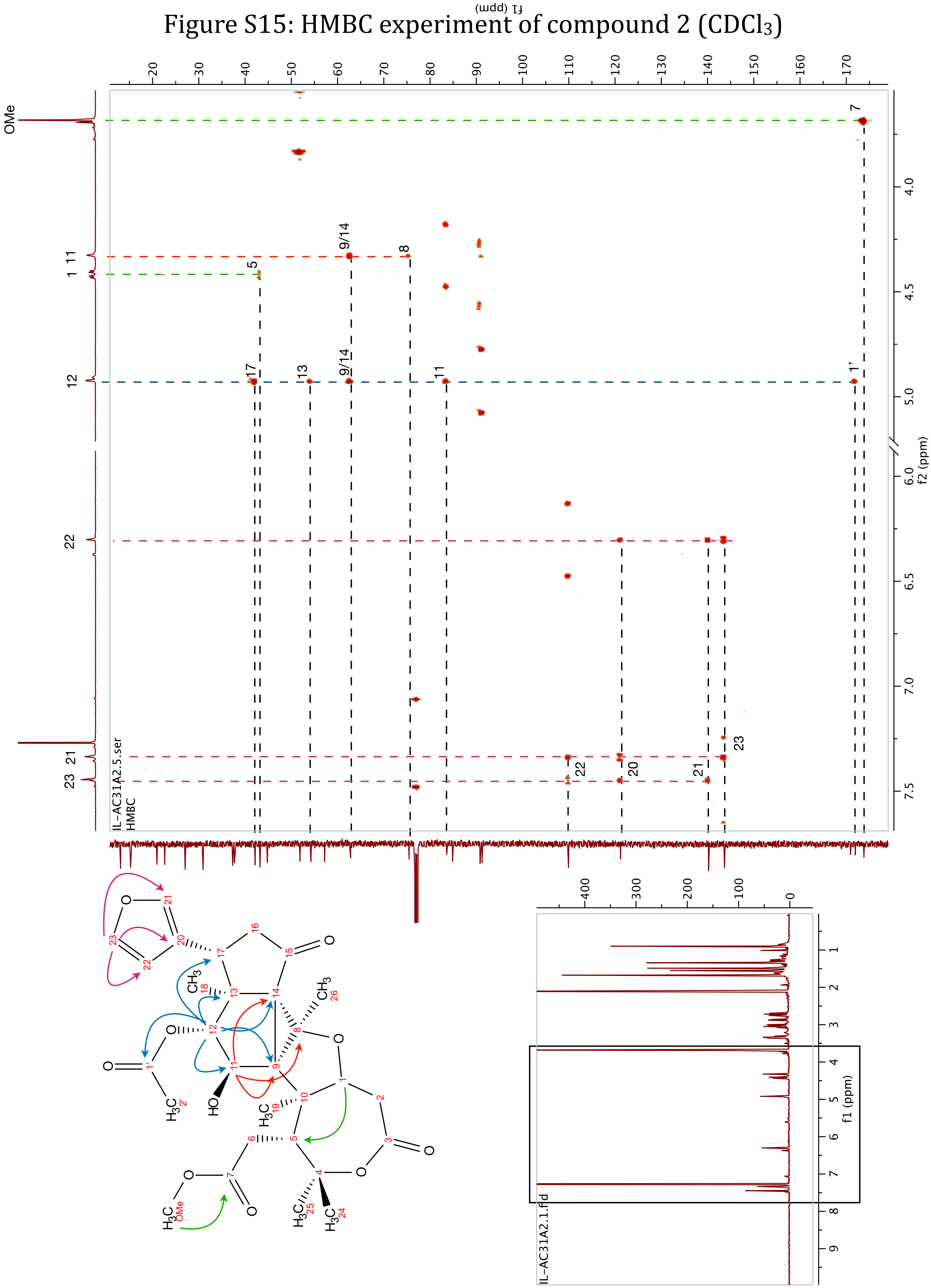

# Figure S16: HMBC experiment of compound 2 ($CDCl_3$)

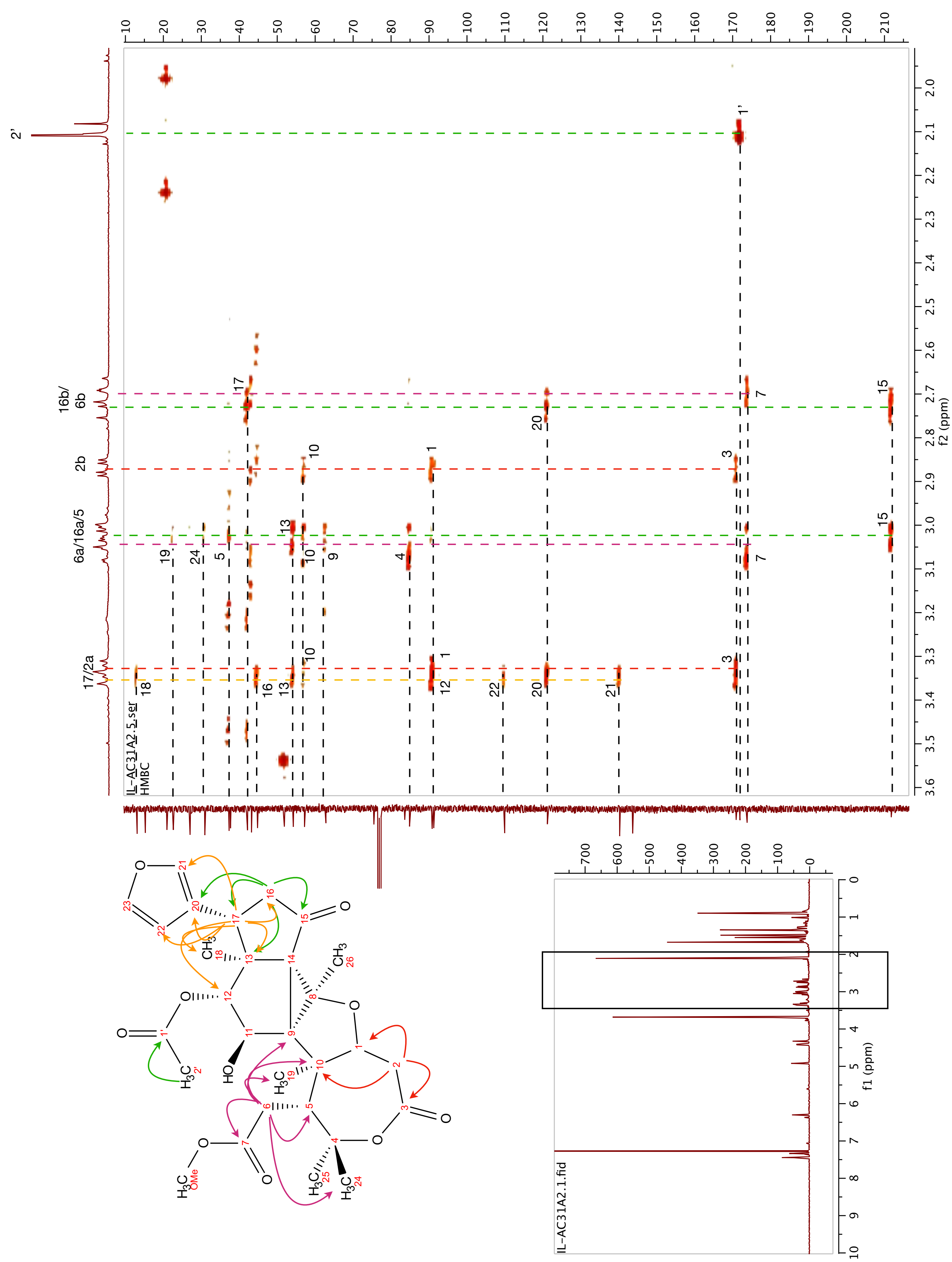

# Figure S17: HMBC experiment of compound 2 ($CDCl_3$)

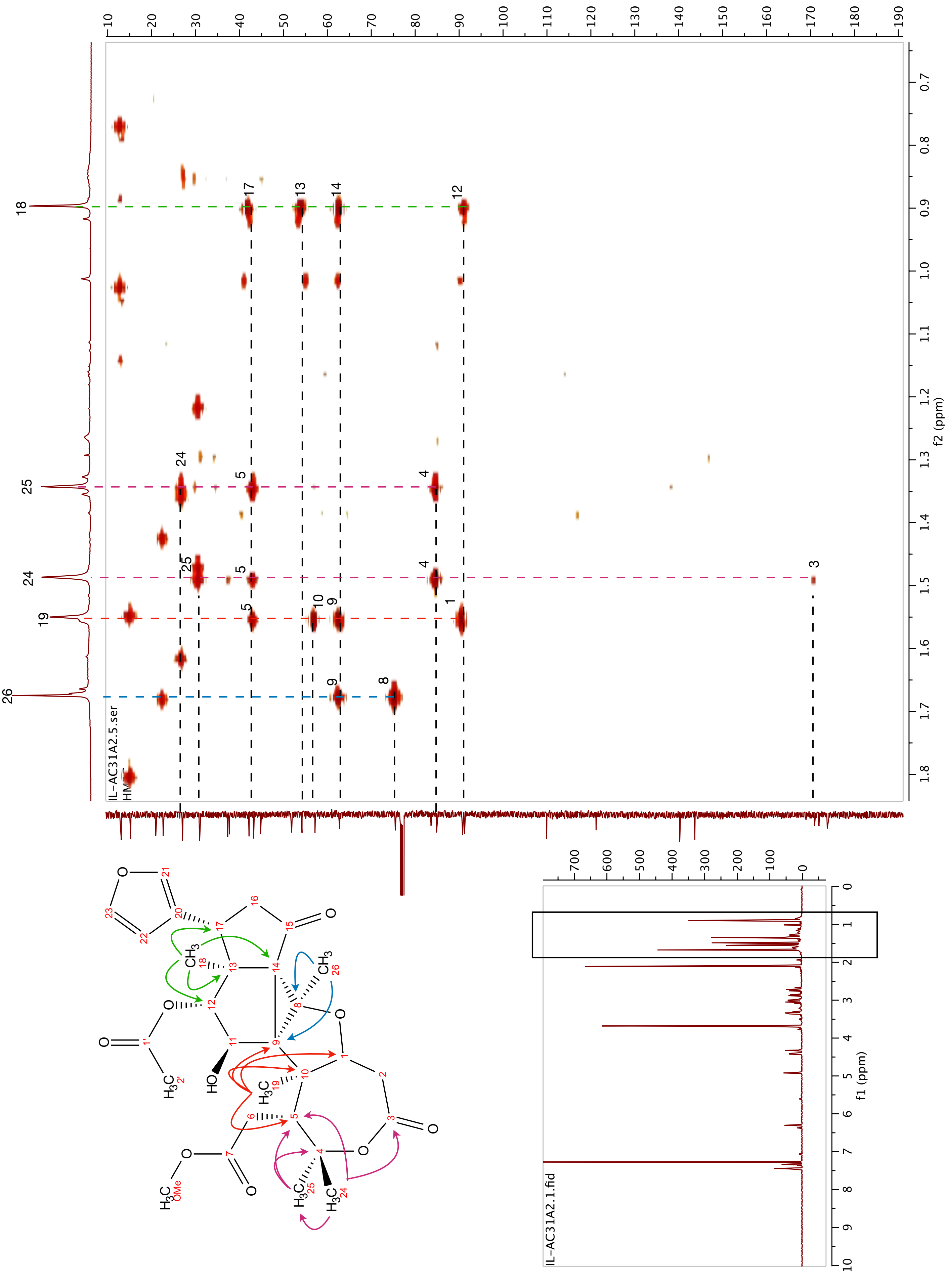

Figure S18: HRMS and IR spectra of compound 2

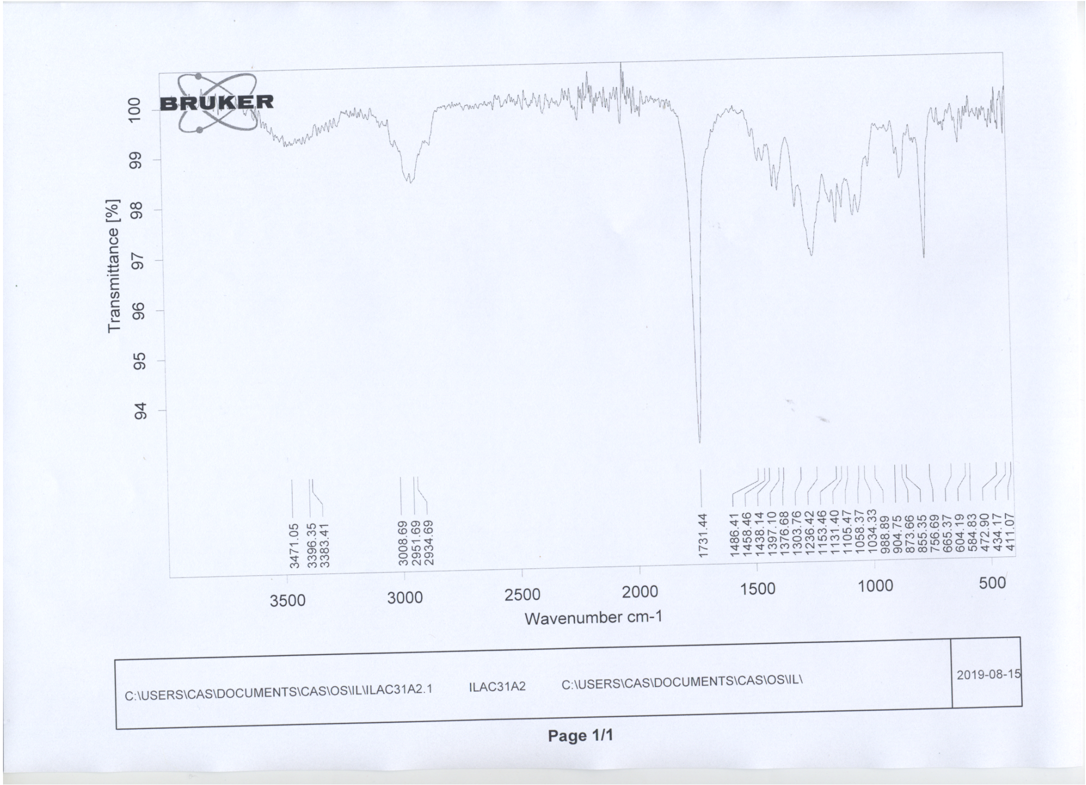


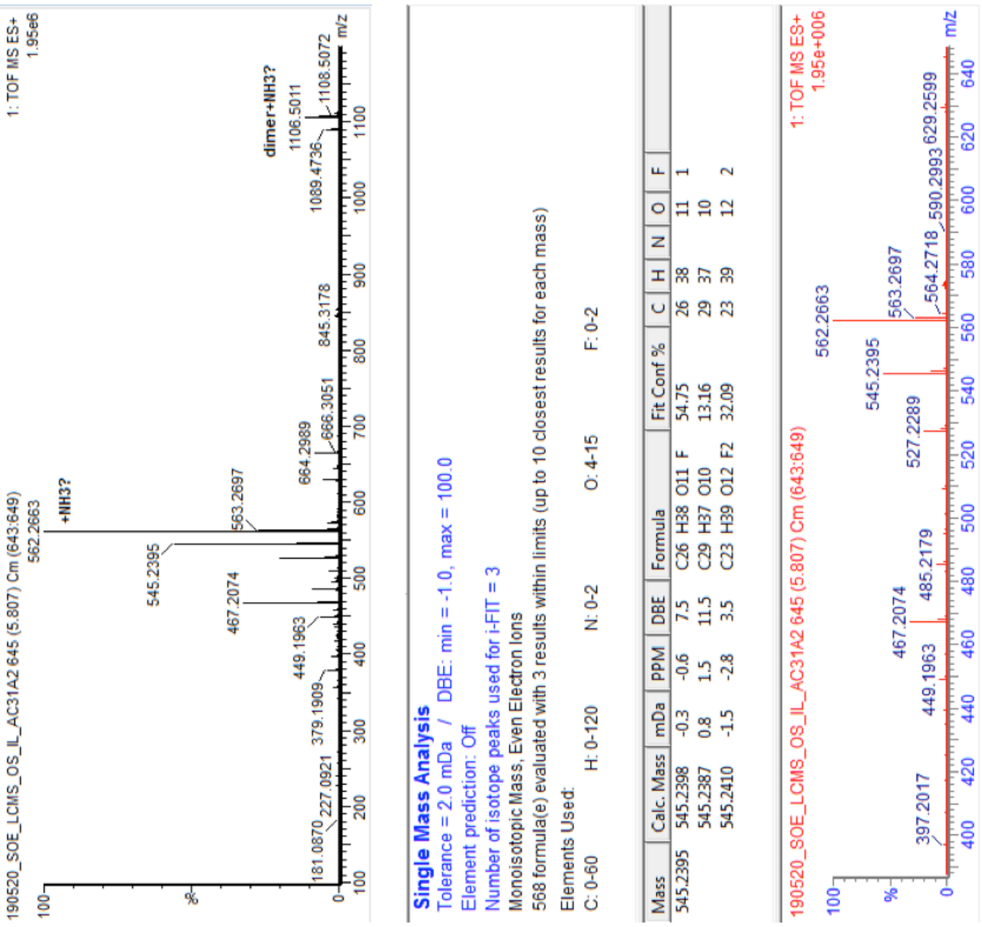


| Mass | Calc. Mass | mDa | PPM | DBE | Formula | Fit Conf % | C | H | N | O | F |
|---|---|---|---|---|---|---|---|---|---|---|---|
| 545.2395 | 545.2398 | -0.3 | -0.6 | 7.5 | C26 H38 O11 F | 54.75 | 26 | 38 | | 11 | 1 |
| | 545.2387 | 0.8 | 1.5 | 11.5 | C29 H37 O10 | 13.16 | 29 | 37 | | 10 | |
| | 545.2410 | -1.5 | -2.8 | 3.5 | C23 H39 O12 F2 | 32.09 | 23 | 39 | | 12 | 2 |

Figure S19: $^{1}H$ NMR of compound 3 ($CDCl_3$)

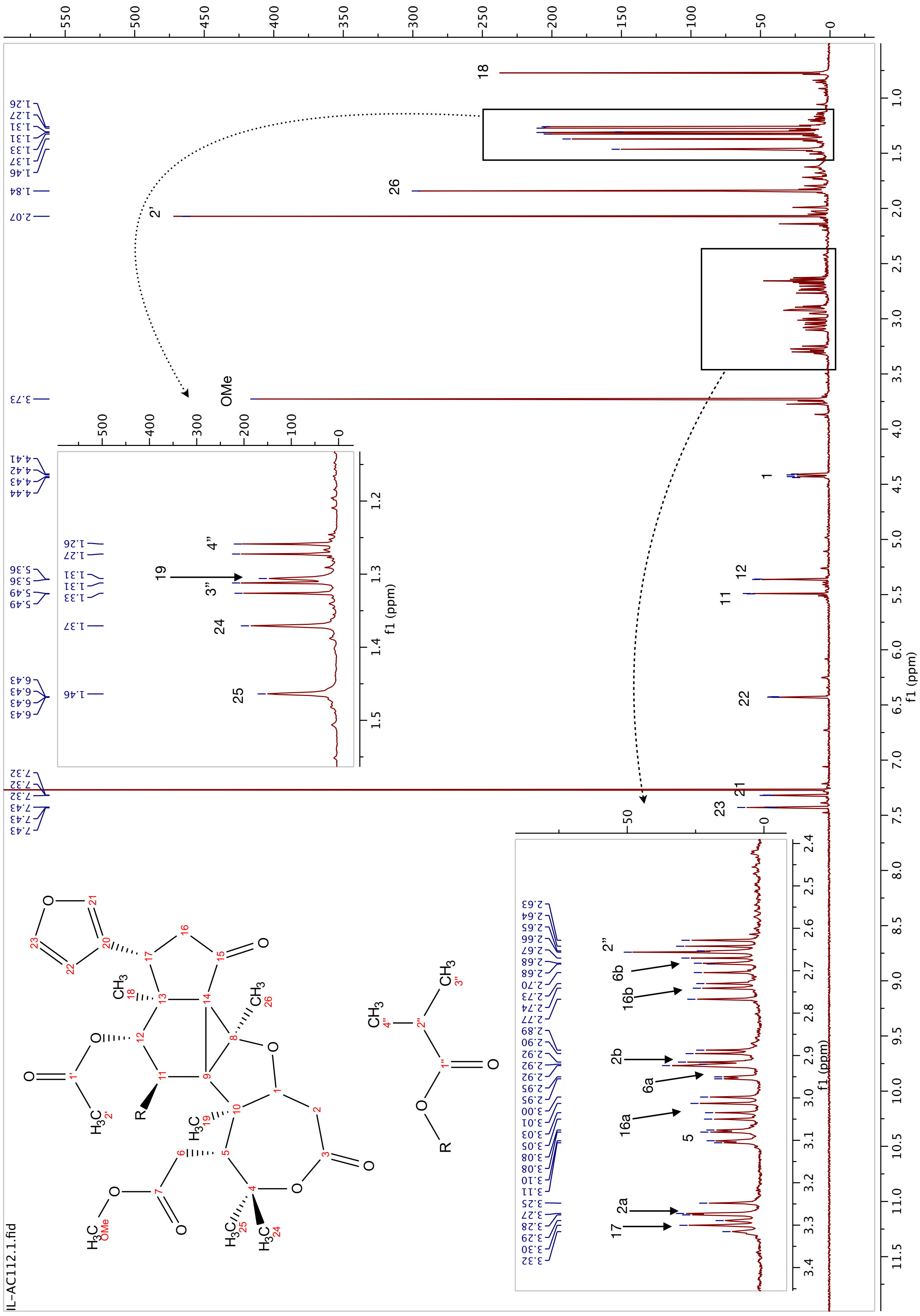

## Figure S20: $^{13}$C NMR of compound 3 ($CDCl_3$)

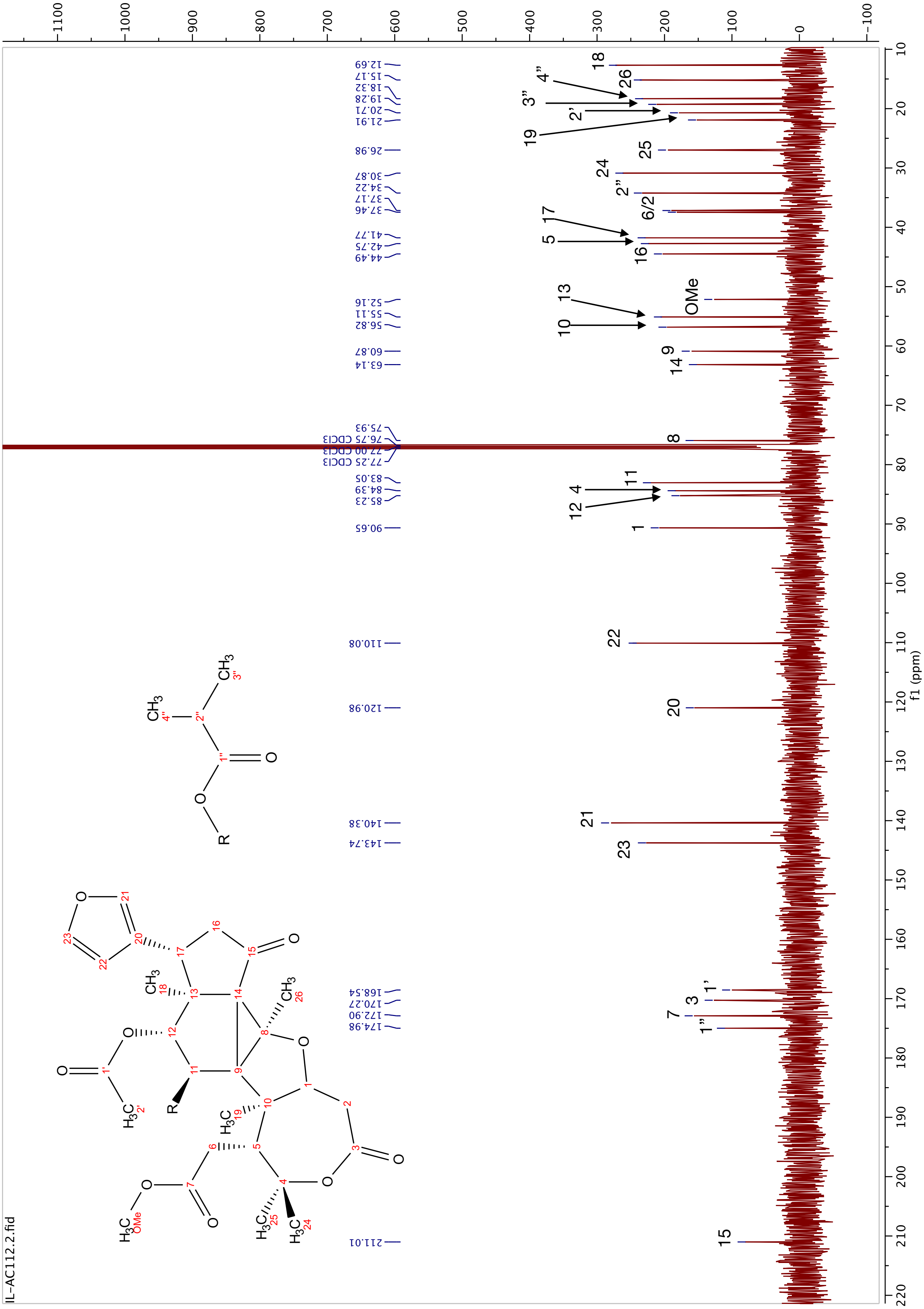

# Figure S21: COSY experiment of compound 3 ($CDCl_3$)

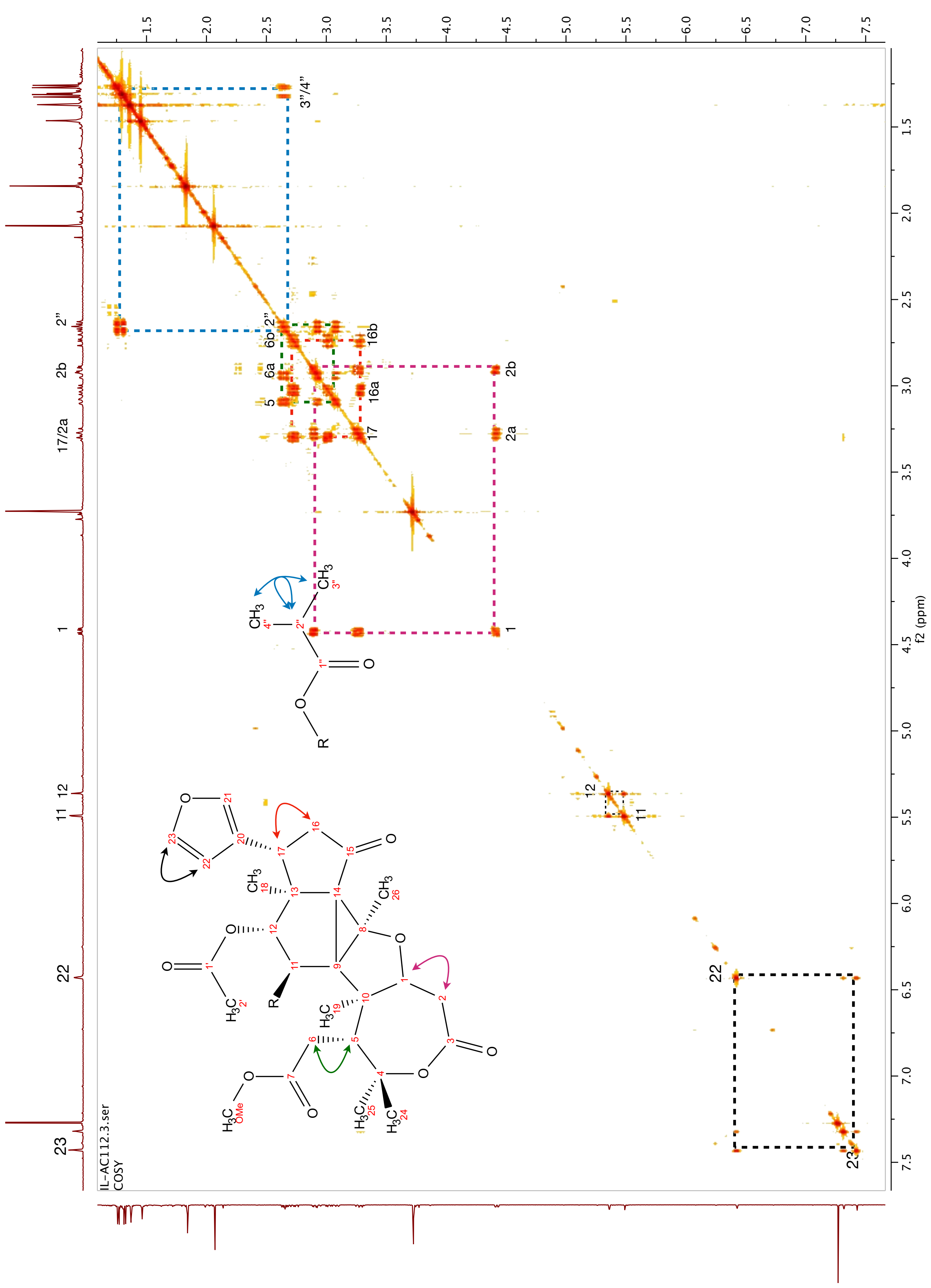

Figure S22: HMQC experiment of compound 3 (CDCl3)

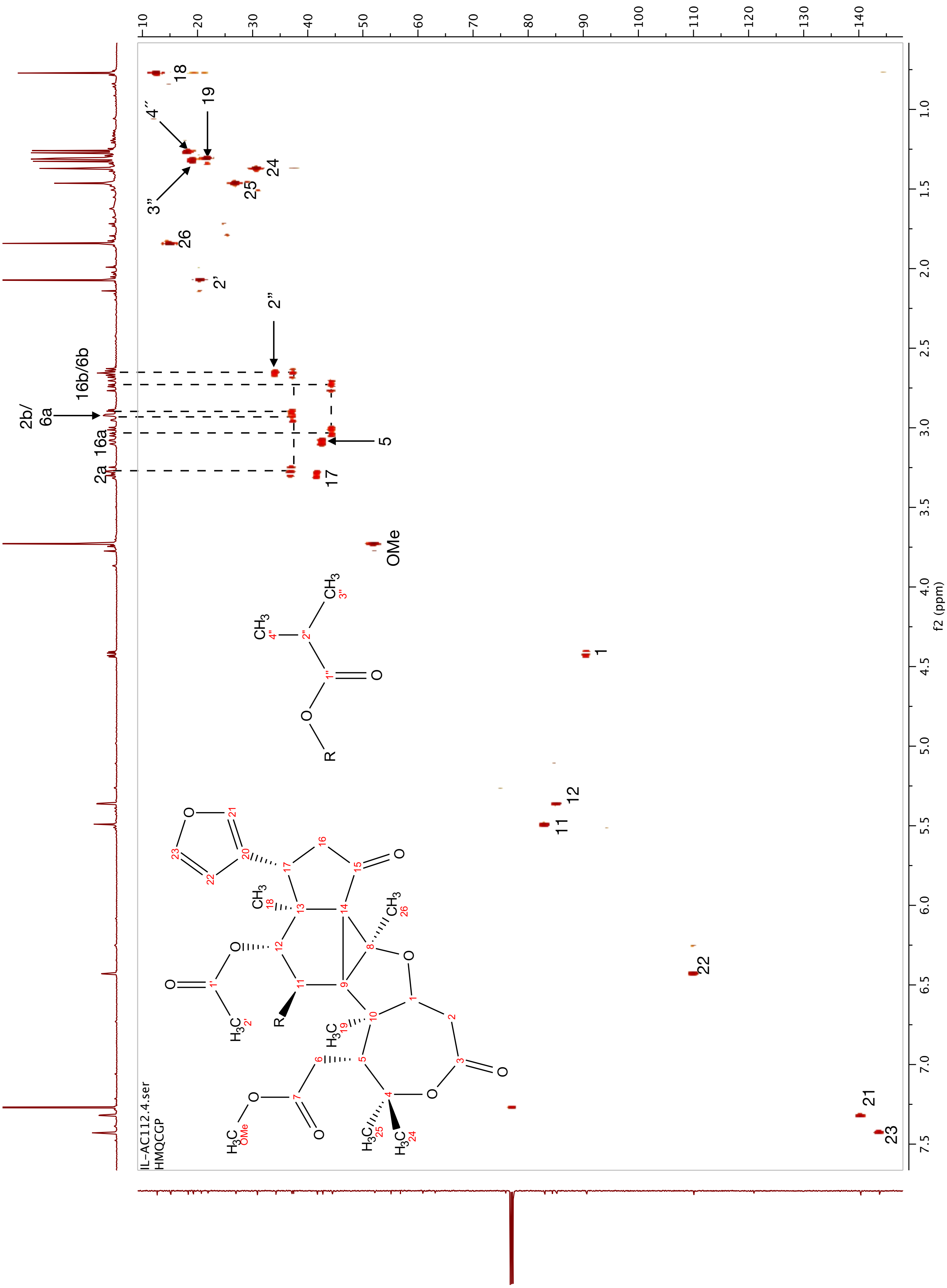

# Figure S23: HMBC experiment of compound 3 ($CDCl_3$)

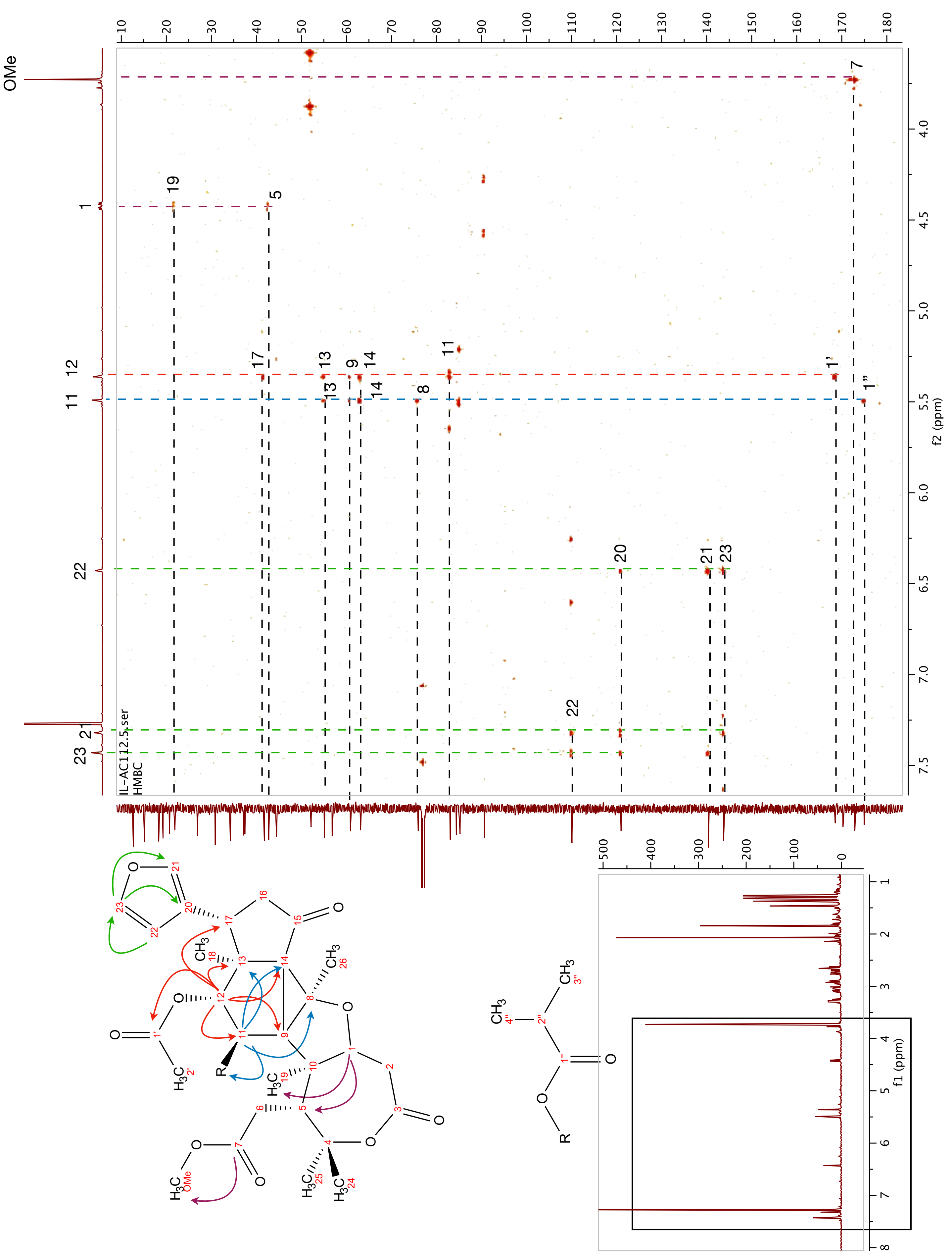

# Figure S24: HMBC experiment of compound 3 ($CDCl_3$)

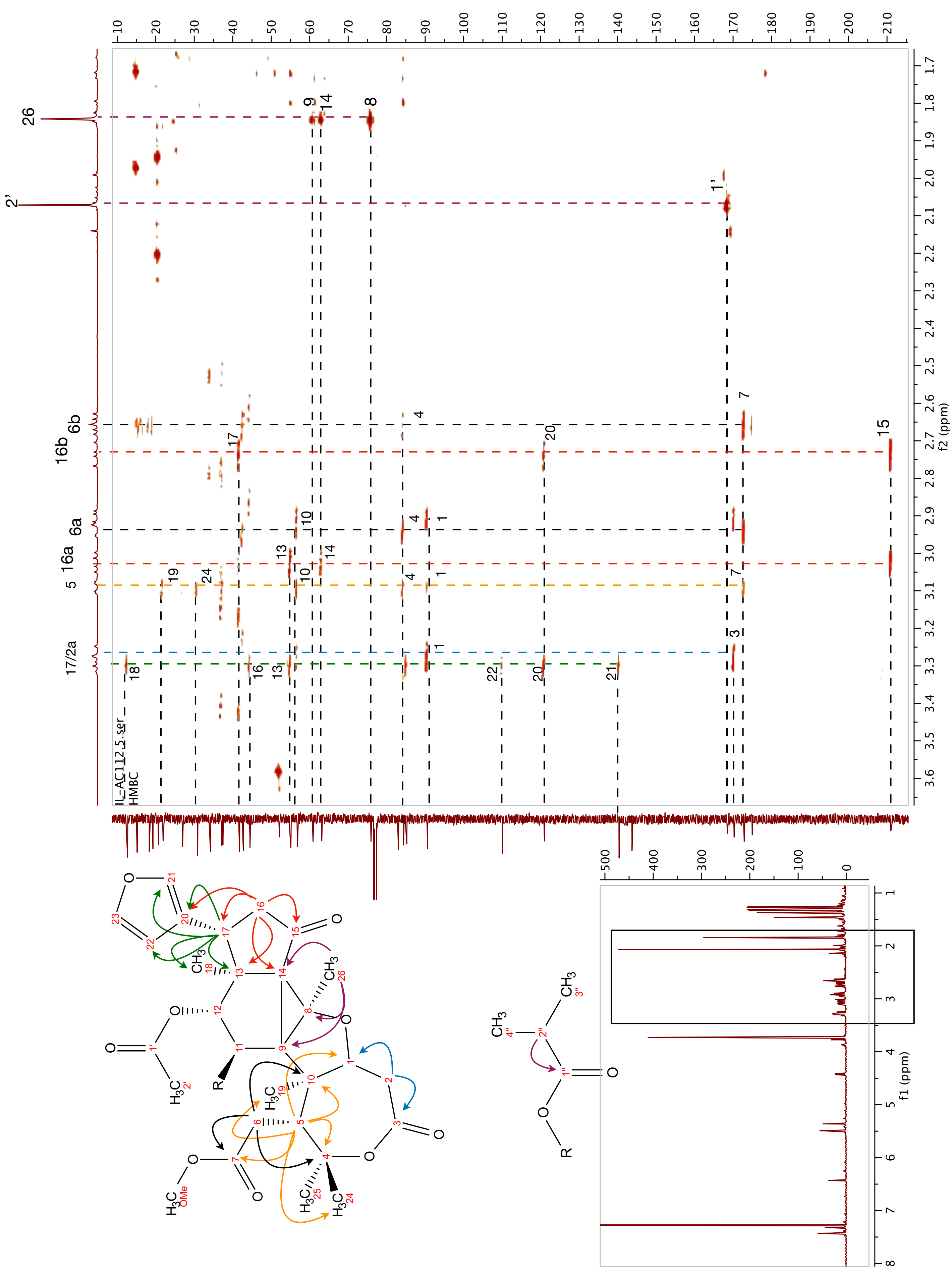

# Figure S25: HMBC experiment of compound 3 ($CDCl_3$)

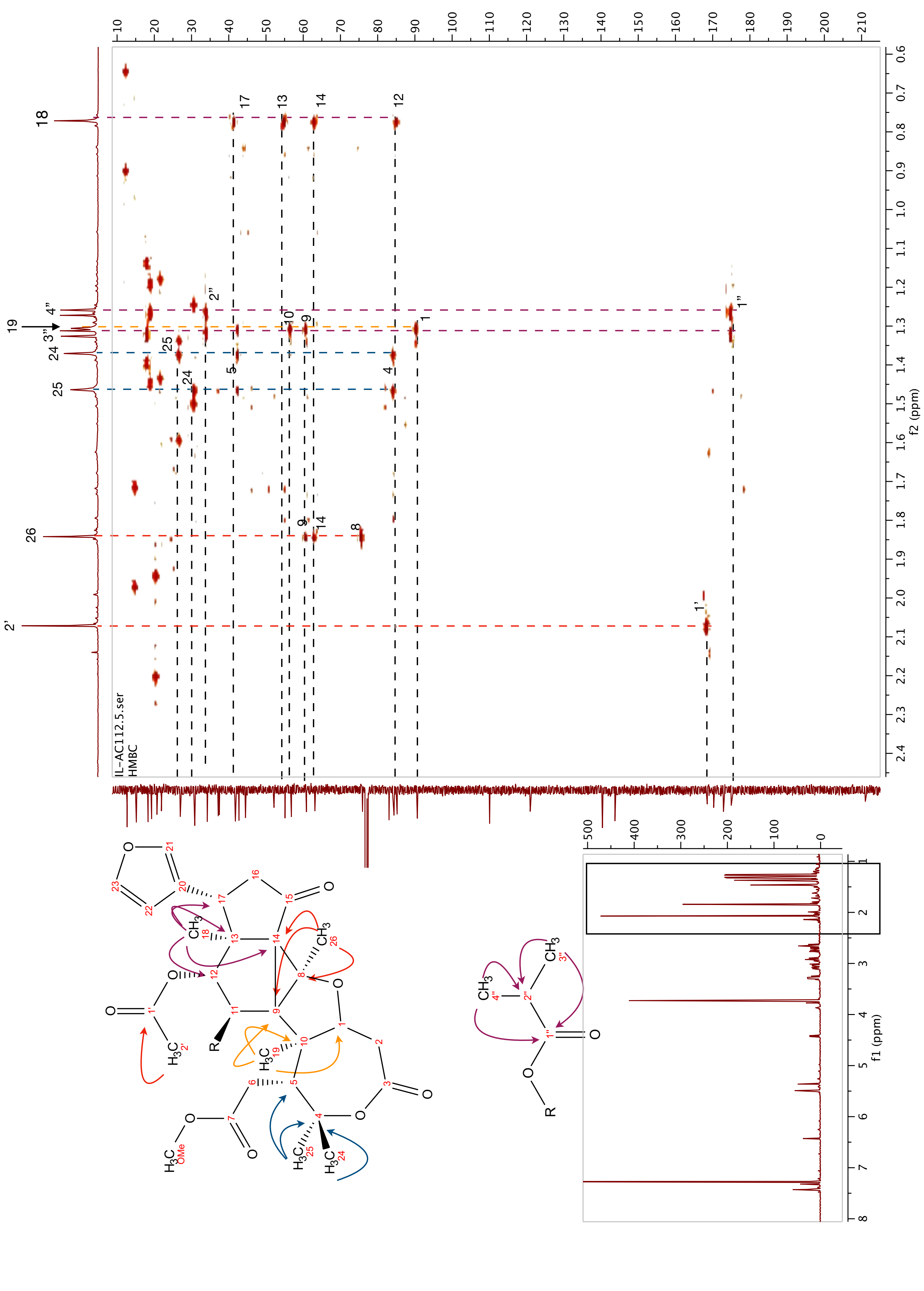

# Figure S26: NOESY experiment of compound 3 ($CDCl_3$)

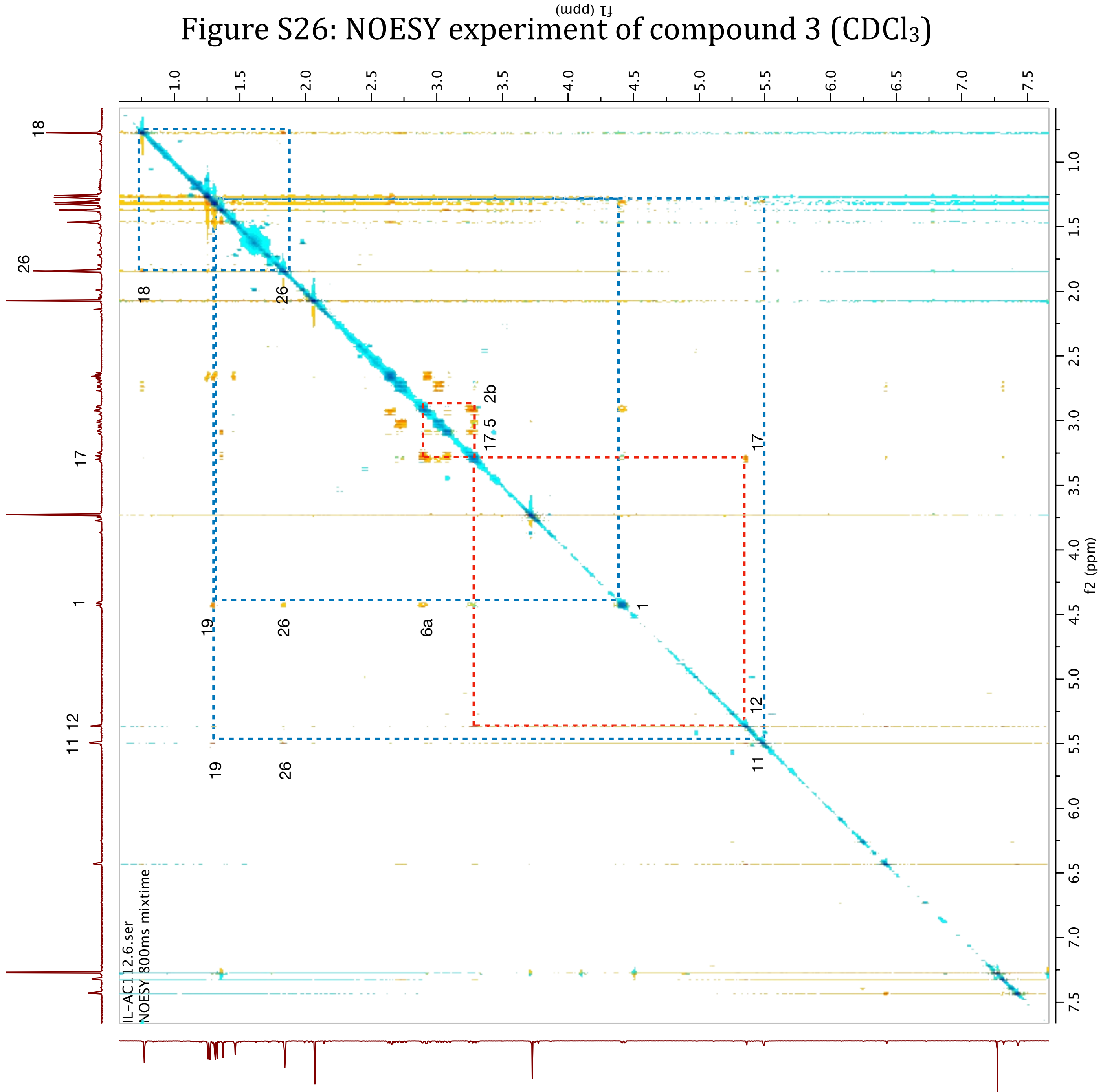


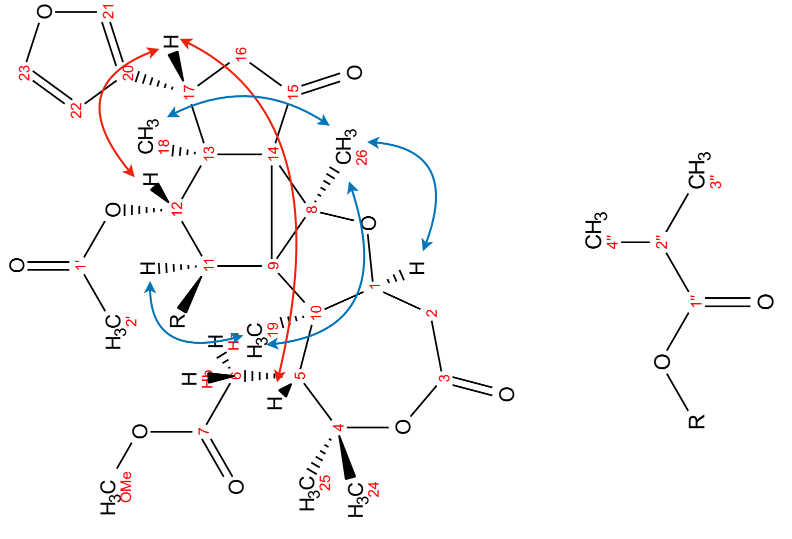

# Figure S27: HRMS and IR spectra of compound 3

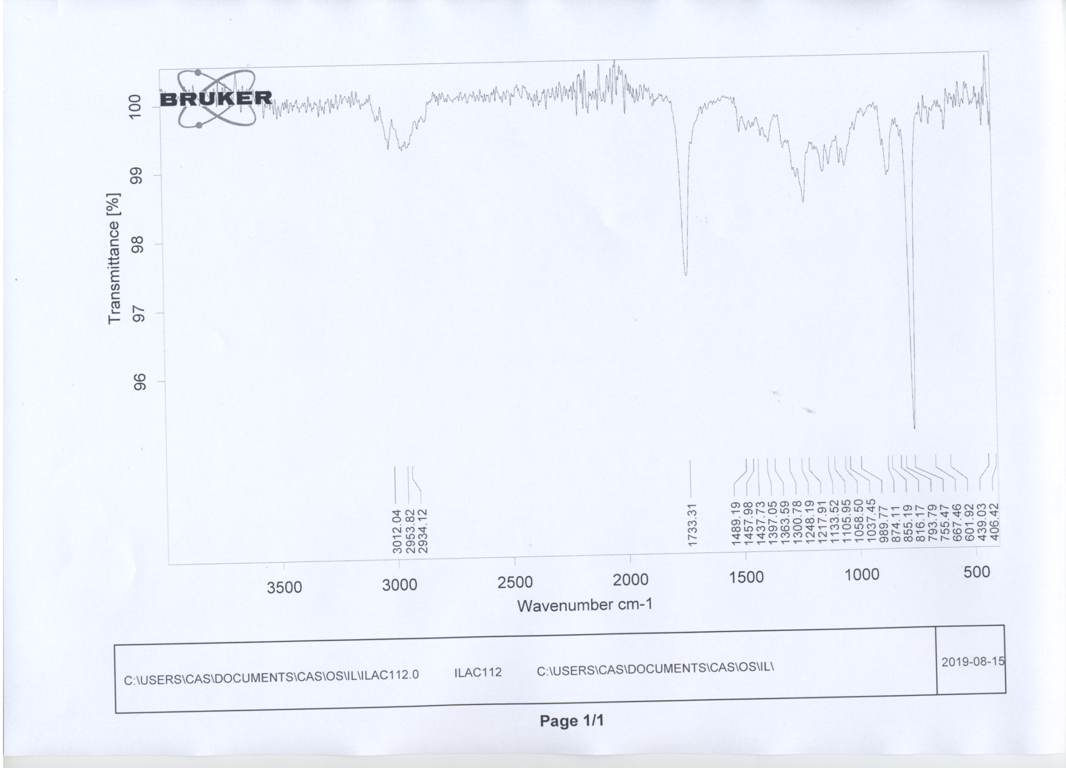


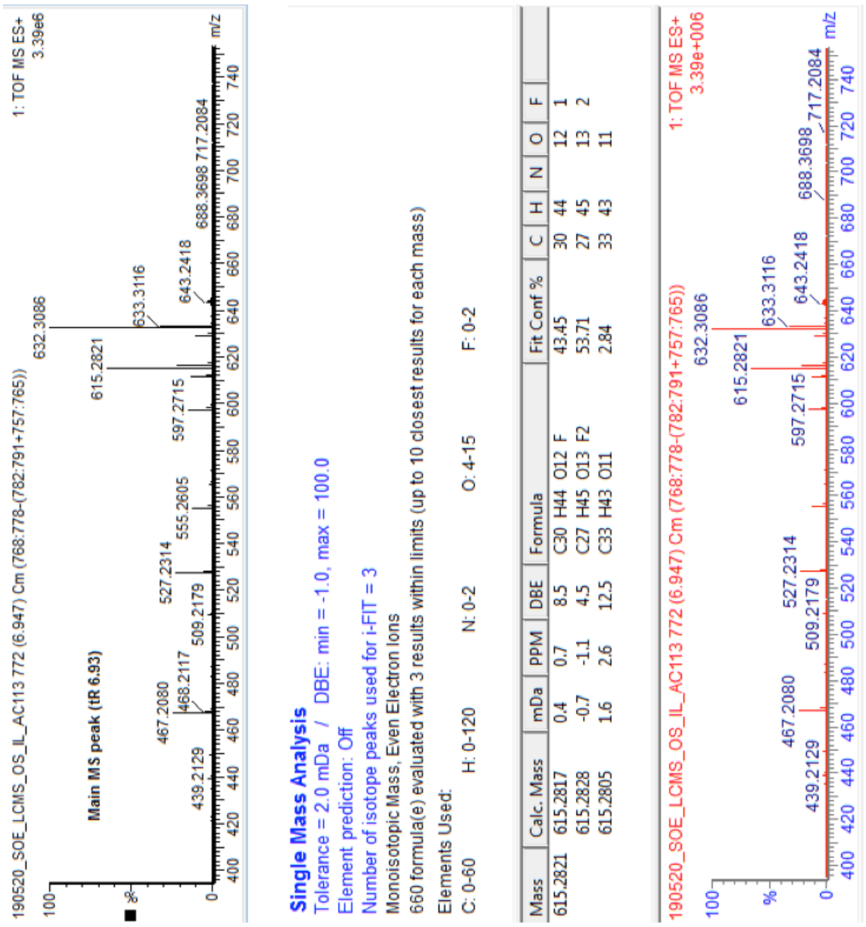

Figure S28: $^{1}$H NMR of compound 4 ($CDCl_3$/500Hz)

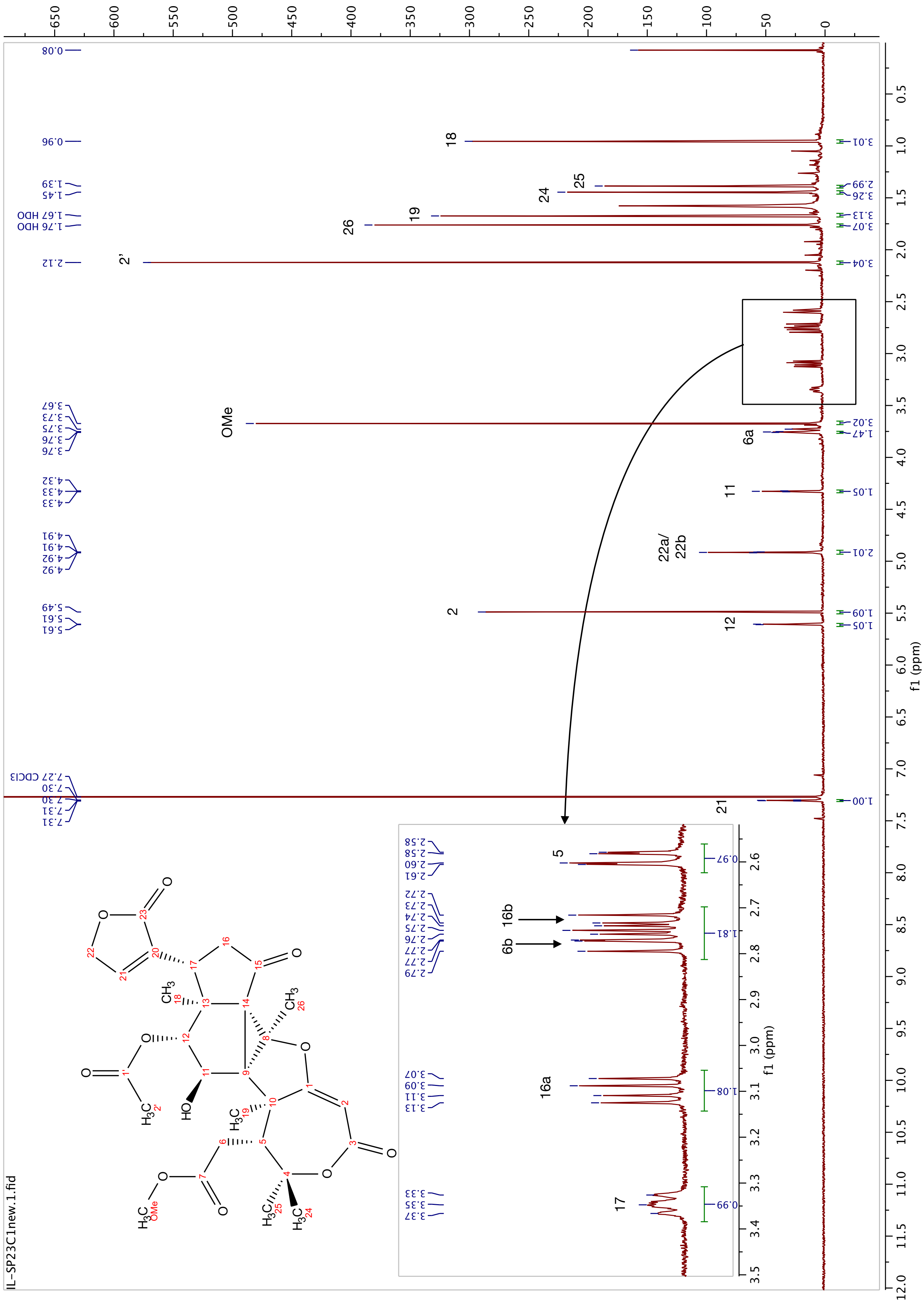

# Figure S29: 13C NMR of compound 4 (CDCl3/500Hz)

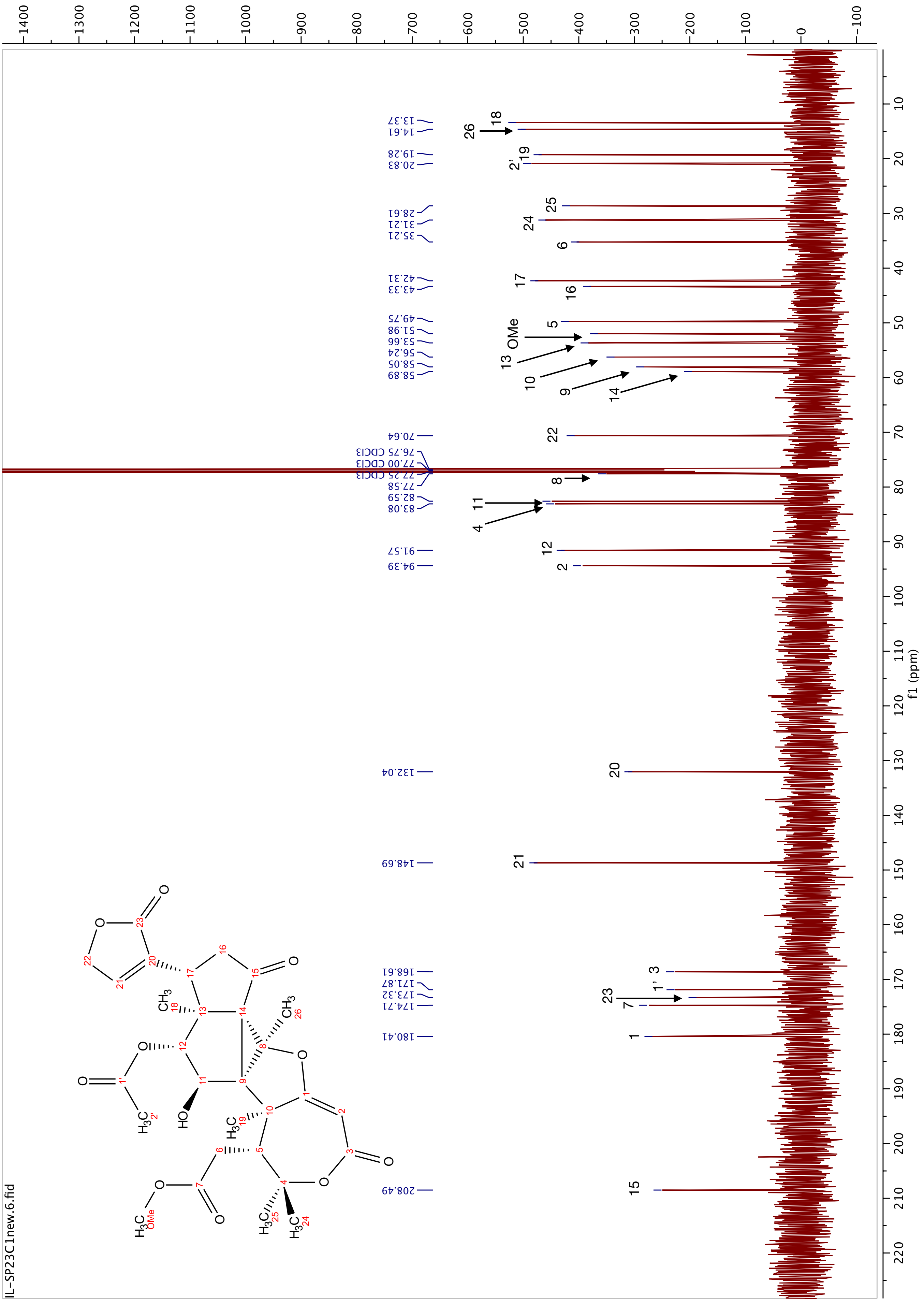

Figure S30: COSY experiment of compound 4 ($CDCl_3$)

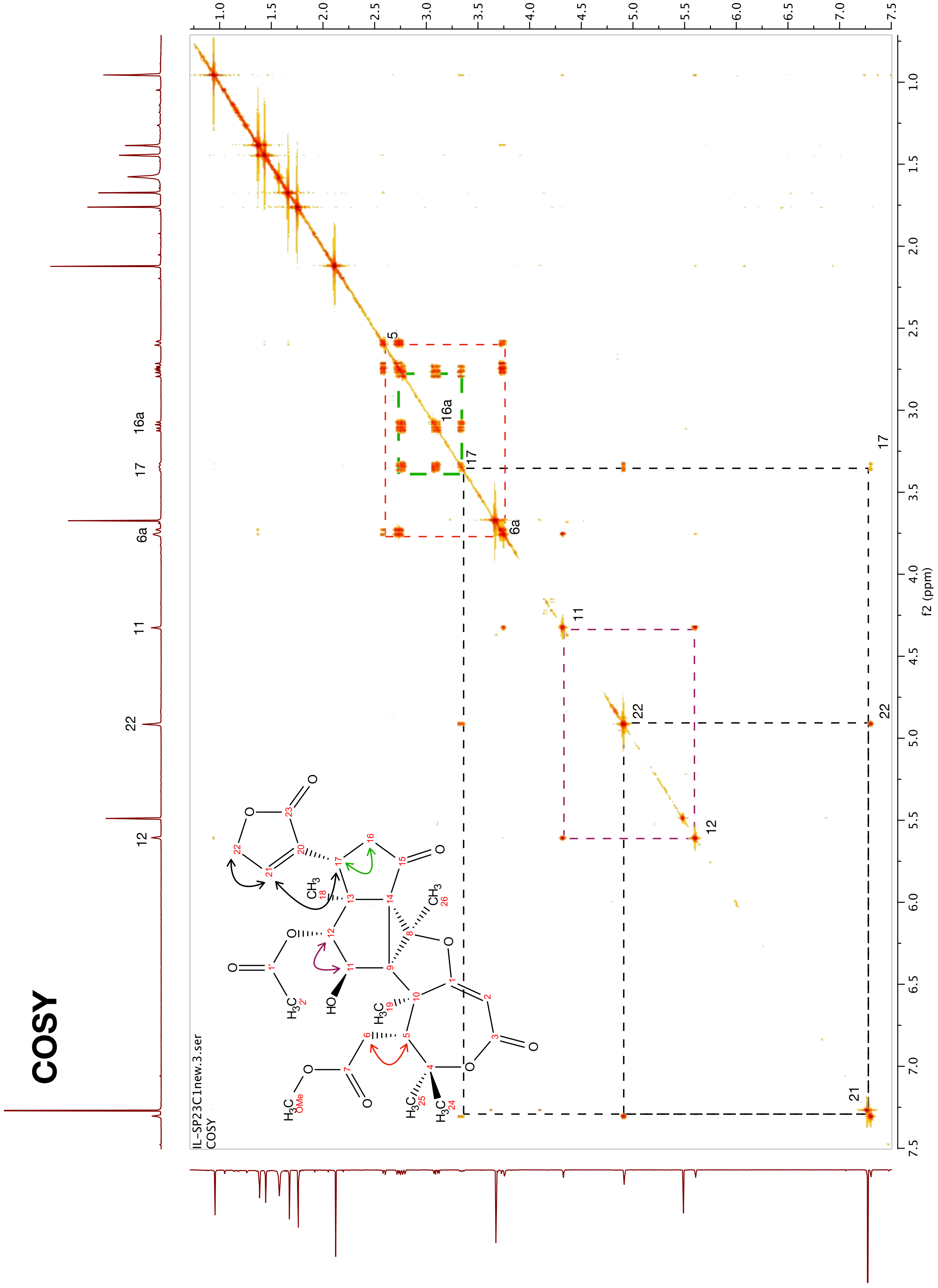

# Figure S31: HMQC experiment of compound 4 (CDCl3)

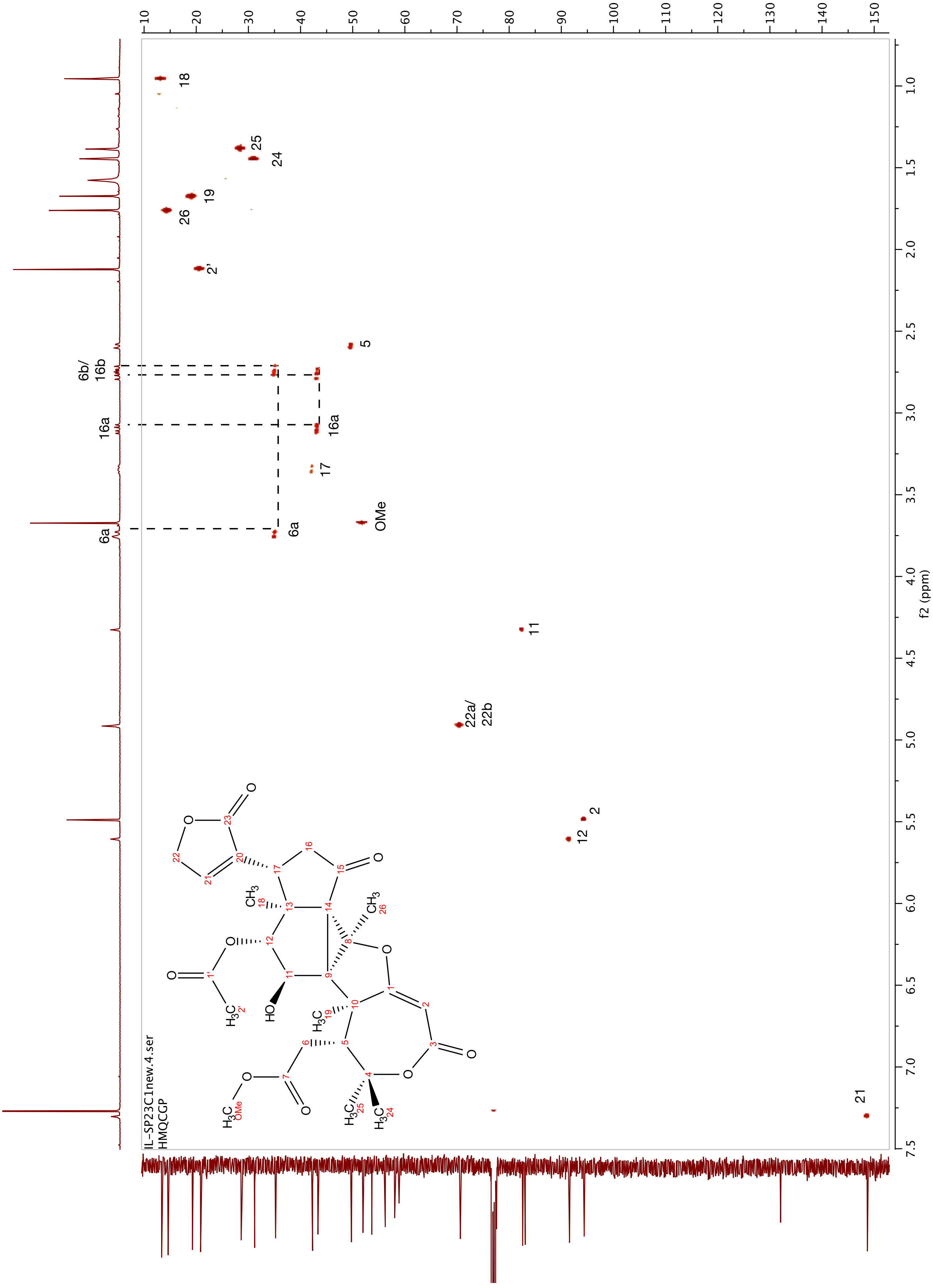

# Figure S32: HMBC experiment of compound 4 ($CDCl_3$)

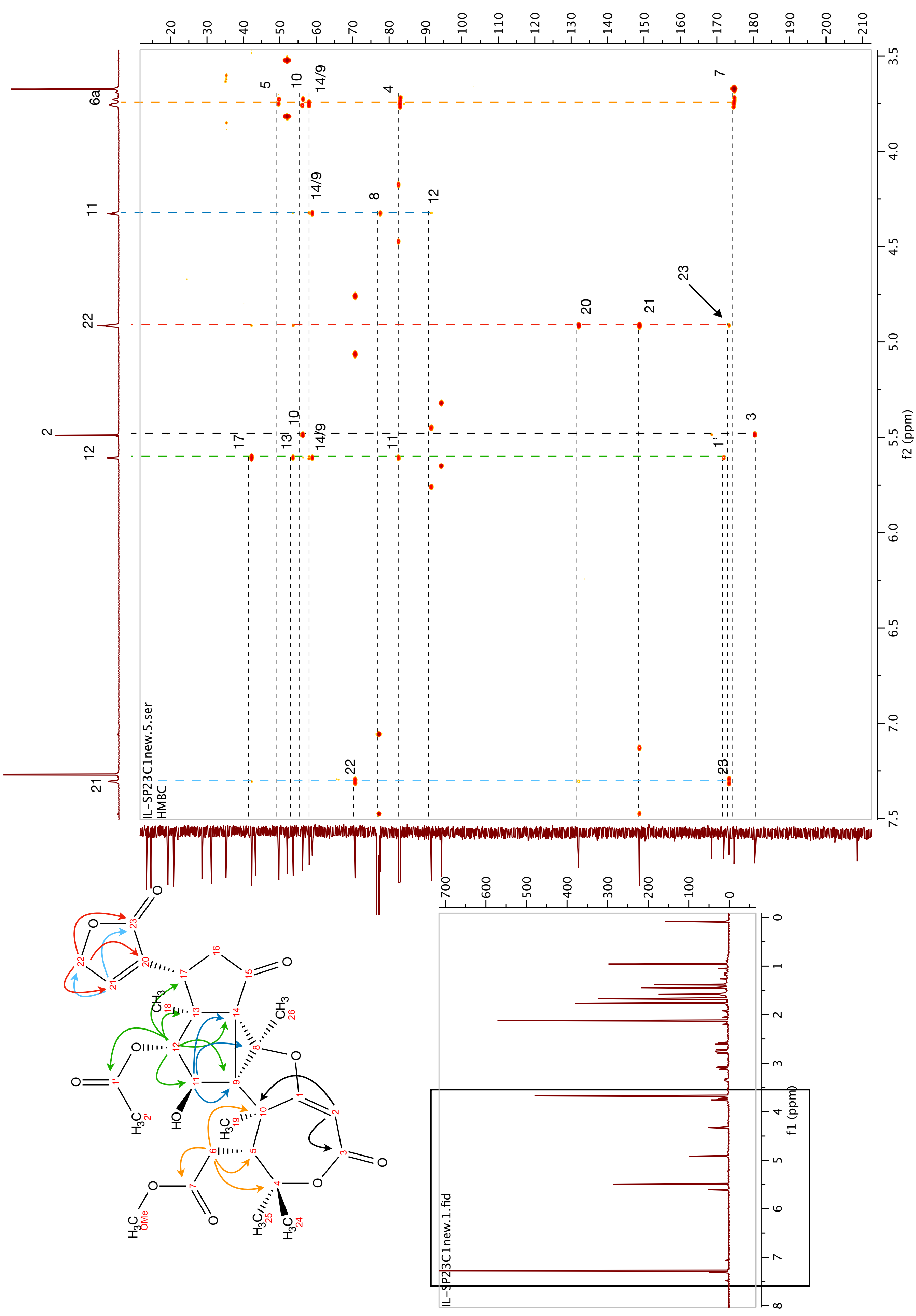

# Figure S33: HMBC experiment of compound 4 ($CDCl_3$)

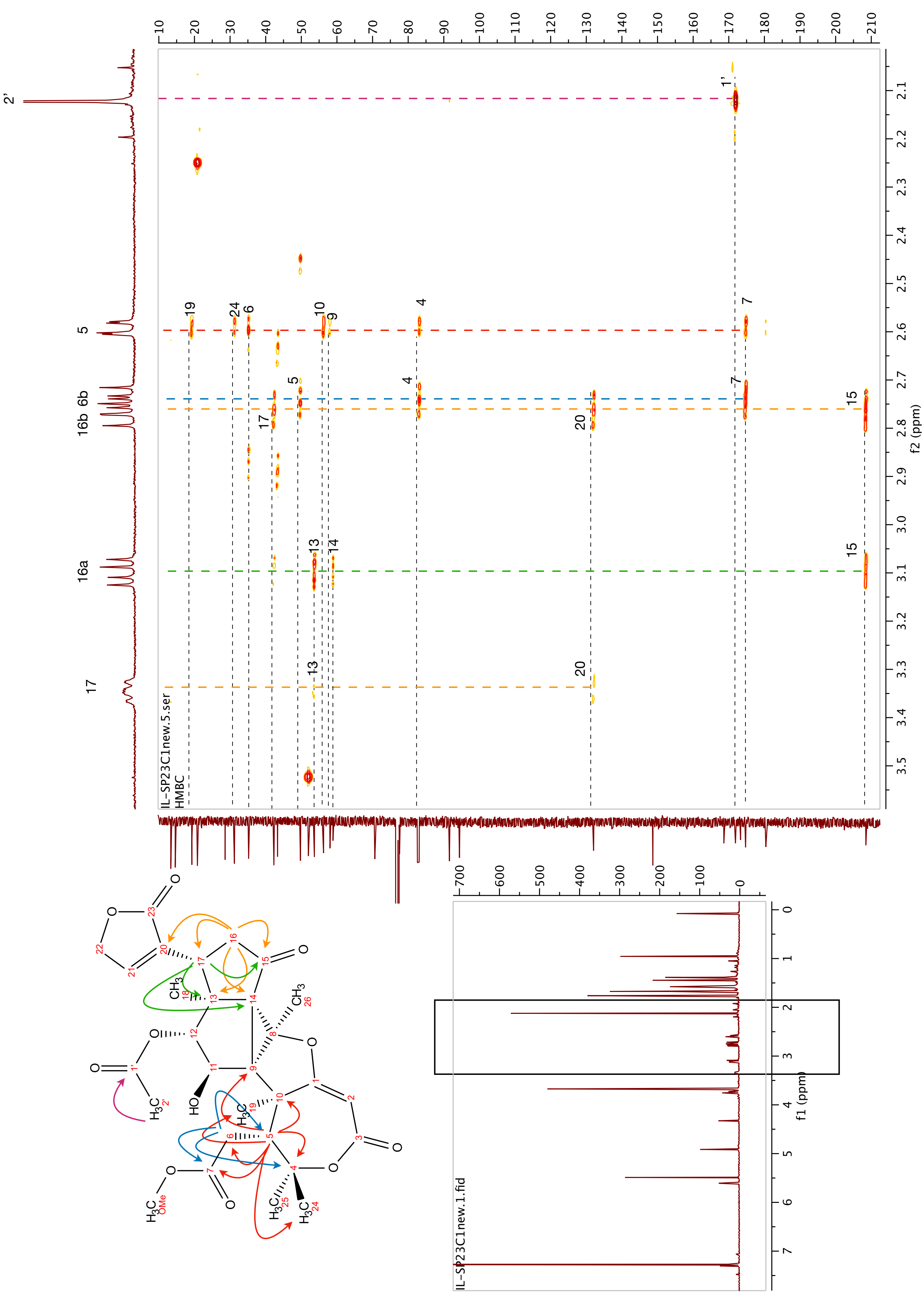

Figure S34: HMBC experiment of compound 4 ($CDCl_3$)

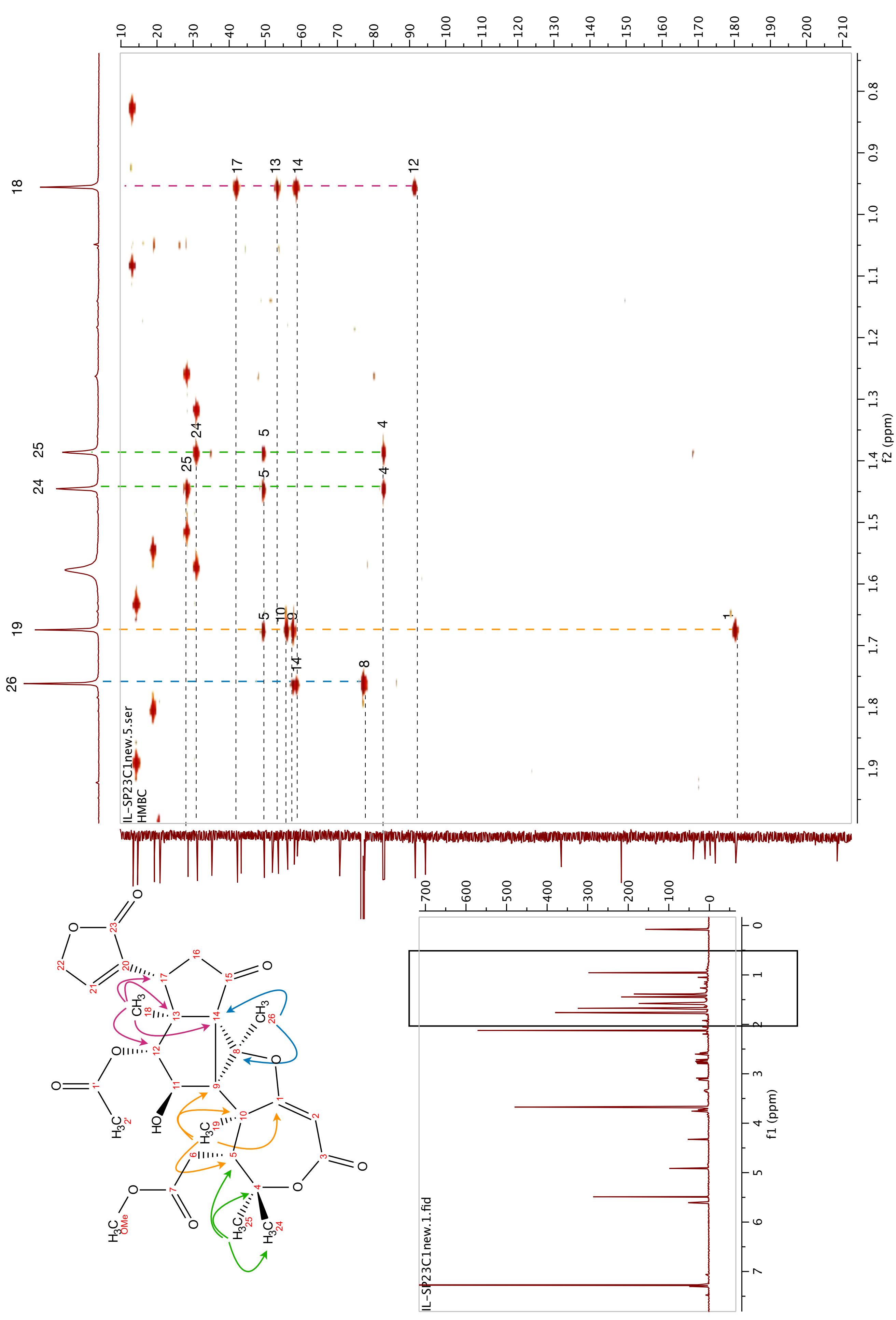

Figure S35: HRMS and IR spectra of compound 4 ($CDCl_3$)

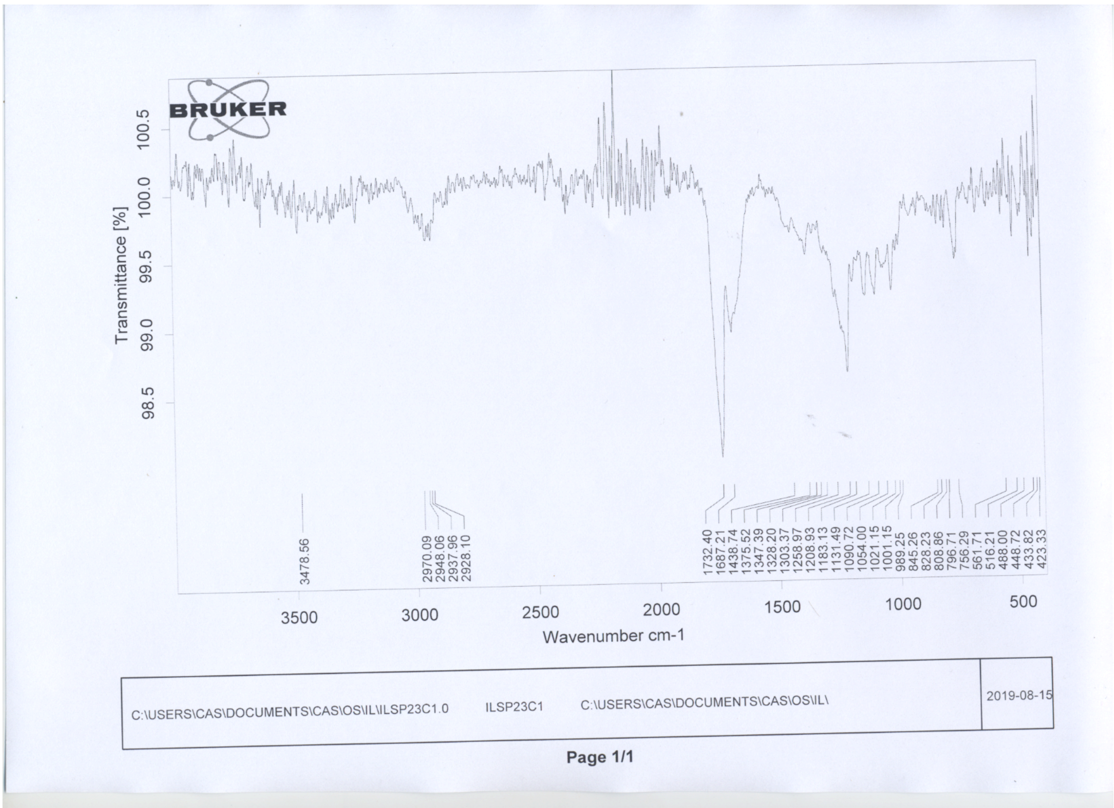


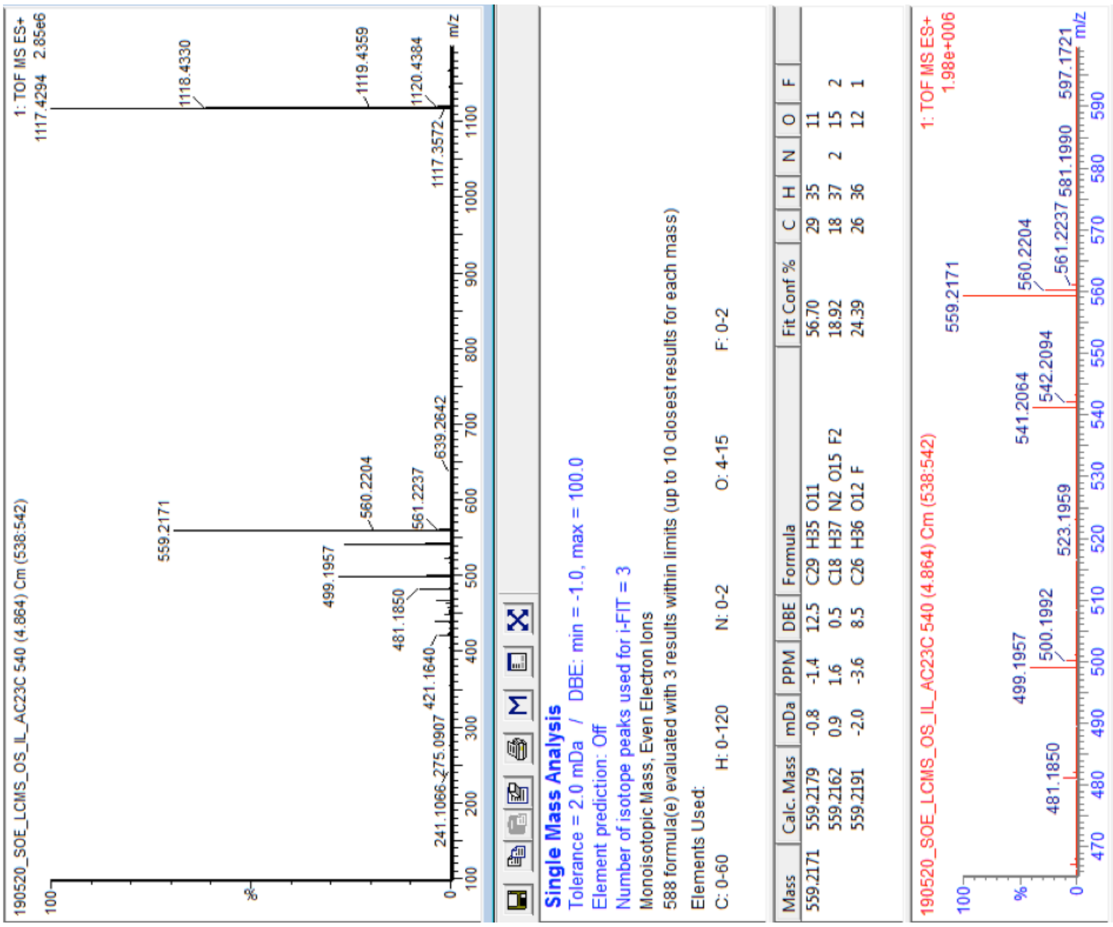

Supplement: Supplementary file 1 [file molecules-26-01019-s001.pdf]
